# Construction of an mRNA-miRNA-lncRNA network prognostic for triple-negative breast cancer

**DOI:** 10.18632/aging.202254

**Published:** 2021-01-03

**Authors:** Yuan Huang, Xiaowei Wang, Yiran Zheng, Wei Chen, Yabing Zheng, Guangliang Li, Weiyang Lou, Xiaojia Wang

**Affiliations:** 1 Department of Breast Medical Oncology, The Cancer Hospital of the University of Chinese Academy of Sciences (Zhejiang Cancer Hospital), Institute of Basic Medicine and Cancer (IBMC), Chinese Academy of Sciences, Zhejiang Province, Hangzhou 310022, China; 2 Department of Colorectal Surgery, Sir Run Run Shaw Hospital, School of Medicine, Zhejiang University, Zhejiang Province, Hangzhou 310016, China; 3 School of Pharmaceutical Sciences, Soochow University, Jiangsu Province, Suzhou 215123, China; 4 Silergy Corporation, Zhejiang Province, Hangzhou 310012, China; 5 Department of Breast Surgery, First Affiliated Hospital, School of Medicine, Zhejiang University, Zhejiang Province, Hangzhou 310003, China

**Keywords:** triple-negative breast cancer, biomarker, competing endogenous RNA, prognosis

## Abstract

The aim of this study was to establish a novel competing endogenous RNA (ceRNA) network able to predict prognosis in patients with triple-negative breast cancer (TNBC). Differential gene expression analysis was performed using the GEO2R tool. Enrichr and STRING were used to conduct protein-protein interaction and pathway enrichment analyses, respectively. Upstream lncRNAs and miRNAs were identified using miRNet and mirTarBase, respectively. Prognostic values, expression, and correlational relationships of mRNAs, lncRNAs, and miRNAs were examined using GEPIA, starBase, and Kaplan-Meier plotter. It total, 860 upregulated and 622 downregulated differentially expressed mRNAs were identified in TNBC. Ten overexpressed and two underexpressed hub genes were screened. Next, 10 key miRNAs upstream of these key hub genes were predicted, of which six upregulated miRNAs were significantly associated with poor prognosis and four downregulated miRNAs were associated with good prognosis in TNBC. NEAT1 and MAL2 were selected as key lncRNAs. An mRNA-miRNA-lncRNA network in TNBC was constructed. Thus, we successfully established a novel mRNA-miRNA-lncRNA regulatory network, each component of which is prognostic for TNBC.

## INTRODUCTION

Triple-negative breast cancer (TNBC) is immunohistochemically defined as estrogen receptor (ER)-negative, progesterone receptor (PR)-negative, and human epidermal growth factor receptor 2 (HER2) nonamplified breast cancer. TNBC composes nearly 15% of all breast cancers [1]. TNBC is aggressive and has the worst prognosis among all breast cancer subtypes. Despite significant advances in breast cancer treatment, TNBC still has a higher relapse rate, shorter overall survival (OS), and limited therapeutic options compared with other subtypes. The median OS for advanced TNBC is about 1 year [2–4]. The molecular mechanisms involved in the occurrence and development of TNBC are still not clear. Thus, it is imperative to determine the mechanisms of TNBC to uncover valid treatment avenues and new prognostic biomarkers.

Numerous studies have revealed that competitive endogenous RNAs (ceRNAs) are vital molecules that regulate a variety of pathological processes [5–7]. The hypothesis of ceRNA, proposed by Salmena et al. [7], suggests a distinct molecular regulatory mechanism for posttranscriptional regulation. The key noncoding RNAs in this hypothesis are miRNAs, which are usually negative regulators of gene expression. ceRNAs can crosstalk by competing for microRNA binding and form a regulatory network across the transcriptome. Recently, it has been reported that the lncRNA-miRNA-mRNA ceRNA network might be a crucial factor in carcinogenesis and cancer development [8–10]. Through microRNA response elements (MREs), lncRNAs act as ceRNAs that sponge miRNA, thereby influencing gene expression of targeted mRNAs. However, the clinical significance of an lncRNA-miRNA-mRNA ceRNA network in TNBC has not been investigated.

In this study, we examined differentially expressed mRNAs (DE-mRNAs) in TNBC tissues, other breast cancer subtype tissues, and normal tissues by mining two Gene Expression Omnibus (GEO) data sets (GSE45827 and GSE6519). Functional enrichment analysis was conducted for these common DE-mRNAs. Next, we performed a protein-protein interaction (PPI) analysis to identify the hub genes. Combining the results of the expression analysis and prognosis analysis for hub genes in breast cancer, we selected 10 upregulated genes and two downregulated genes for further study. We then identified potential upstream lncRNAs and miRNAs and assessed the expression and prognostic value of these lncRNAs and miRNAs in breast cancer. The correlations between mRNAs, miRNAs, and lncRNAs were investigated according to the ceRNA hypothesis. Thus, we established a novel lncRNA-miRNA-mRNA ceRNA network, the components of which can be used to predict prognosis or serve as treatment targets in TNBC.

## RESULTS

### Identification of candidate DE-mRNAs in TNBC

**Figure 1:**
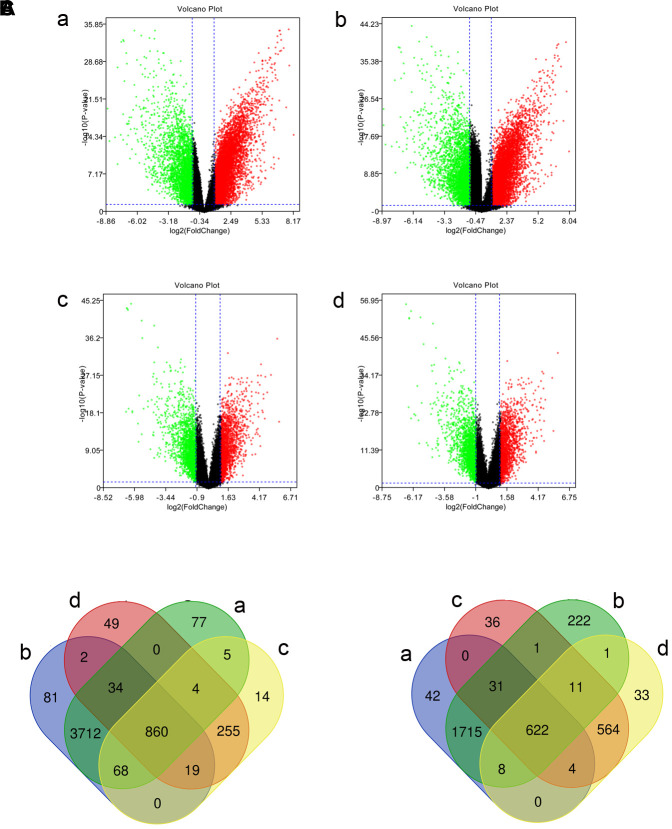
**Identified differentially expressed mRNAs (DE-mRNAs) among triple-negative breast cancer (TNBC) tissues, tissues of other types of breast cancer, and normal samples in two Gene Expression Omnibus data sets.** (**A**) The volcano plots of DE-mRNAs in the GSE45827 and GSE65194 data sets. The x-axis stands for log2 (fold change) of gene expression, and y-axis represents log-transformed *P* value. The red dots and green dots indicate the significantly overexpressed and underexpressed genes, respectively. The black dots indicate genes with no significant differential expression. |log2FC| > 1 and *P* value < 0.05 were the cutoff criteria. (**B**) The intersection of upregulated DE-mRNAs. (**C**) The intersection of downregulated DE-mRNAs. a: TNBC compared with normal samples in GSE45827; b: TNBC compared with normal samples in GSE65194; c: TNBC compared with tissues of other subtypes of breast cancer in GSE45827; d: TNBC compared with tissues of other subtypes of breast cancer in GSE65194.

We screened the gene expression microarrays related to TNBC in the GEO database, and the GSE45827 and GSE65194 data sets were ultimately included. Next, we identified differentially expressed genes between TNBC tissues, tissues of other subtypes of breast cancer, and normal breast tissues using GEO2R (|log2FC| > 1 and *P* value < 0.05). DE-mRNAs in those data sets are shown as volcano plots in Figure 1A. In the GSE45827 data set, 4760 overexpressed mRNAs and 2422 underexpressed mRNAs were identified in the TNBC tissues relative to normal control samples. In addition, 1225 overexpressed and 1269 underexpressed genes were found in TNBC tissues relative to other breast cancer subtype tissues. In the GSE65194 data set, 4776 mRNAs were overexpressed and 2611 mRNAs were underexpressed in TNBC tissues compared with normal control samples. In addition, 1222 genes were overexpressed and 1244 genes were underexpressed in TNBC tissues compared with other breast cancer subtype tissues. After overlapping the genes, 860 upregulated (Figure 1B) and 622 downregulated (Figure 1C) common genes were identified. These DE-miRNAs are listed in Supplementary Table 1 and were chosen for further study.

### GO functional and KEGG pathway enrichment analysis

**Figure 2:**
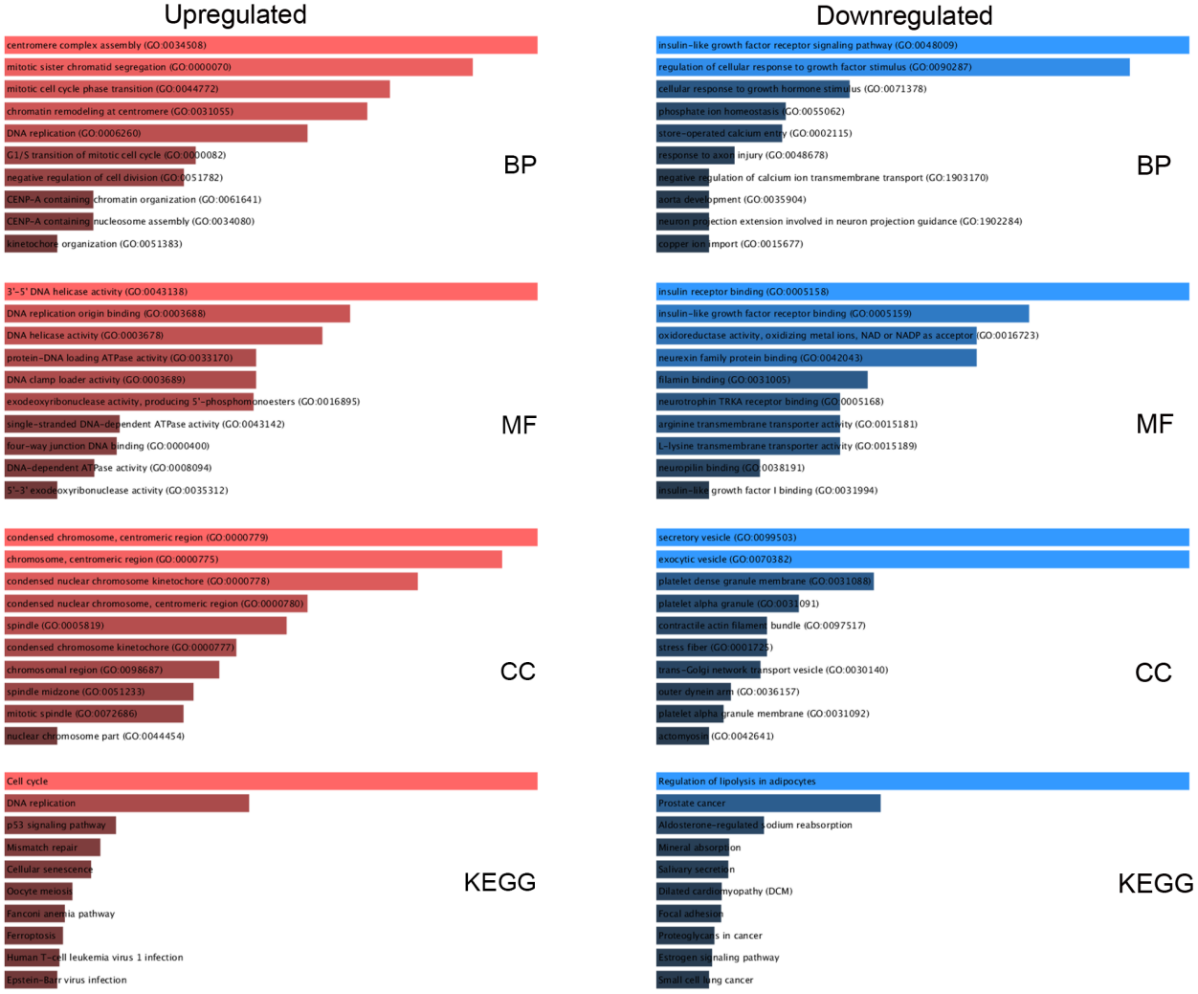
**Functional enrichment analysis for key DE-mRNAs.** The top 10 enriched molecular function (MF), cellular component (CC), biological process (BP), and KEGG pathways of the upregulated and downregulated significant DE-mRNAs.

We performed Gene Ontology (GO) functional annotation and pathway enrichment analysis using the Enrichr database to examine the functions of the DE-mRNAs. GO analysis was conducted as three sublevels: cellular component (CC), biological process (BP), and molecular function (MF). For pathway enrichment, KEGG’s cell signaling pathway was conducted. As shown in Figure 2, overexpressed DE-mRNAs were dramatically enriched in terms of cell division and proliferation, such as centromere complex assembly, mitotic sister chromatid segregation, and DNA replication in the BP category; centromeric region, chromosome, and spindle in the CC group; and DNA helicase activity, DNA replication origin binding, and ATPase activity in the MF category. Besides cell cycle and DNA replication, signaling pathways related to cell death appeared in the top 10 enriched KEGG pathways for upregulated DE-mRNAs, including the p53 signaling pathway, mismatch repair, cellular senescence, and ferroptosis. For downregulated DE-mRNAs, the enriched GO functions included regulation of cellular response to growth factor stimulus, insulin-like growth factor receptor signaling pathway, and phosphate ion homeostasis in the BP category; vesicle, platelet granule membrane, and contractile actin filament bundle in the CC category; and insulin-like growth factor receptor binding, insulin receptor binding, oxidoreductase activity, and transmembrane transporter activity in the MF category. Subsequently, KEGG pathway analysis indicated that these downregulated DE-mRNAs were considerably enriched in several pathways associated with many cancers, such as prostate cancer and small-cell lung cancer, and proteoglycans.

### Establishment of PPI network and detection of hub genes

**Figure 3:**
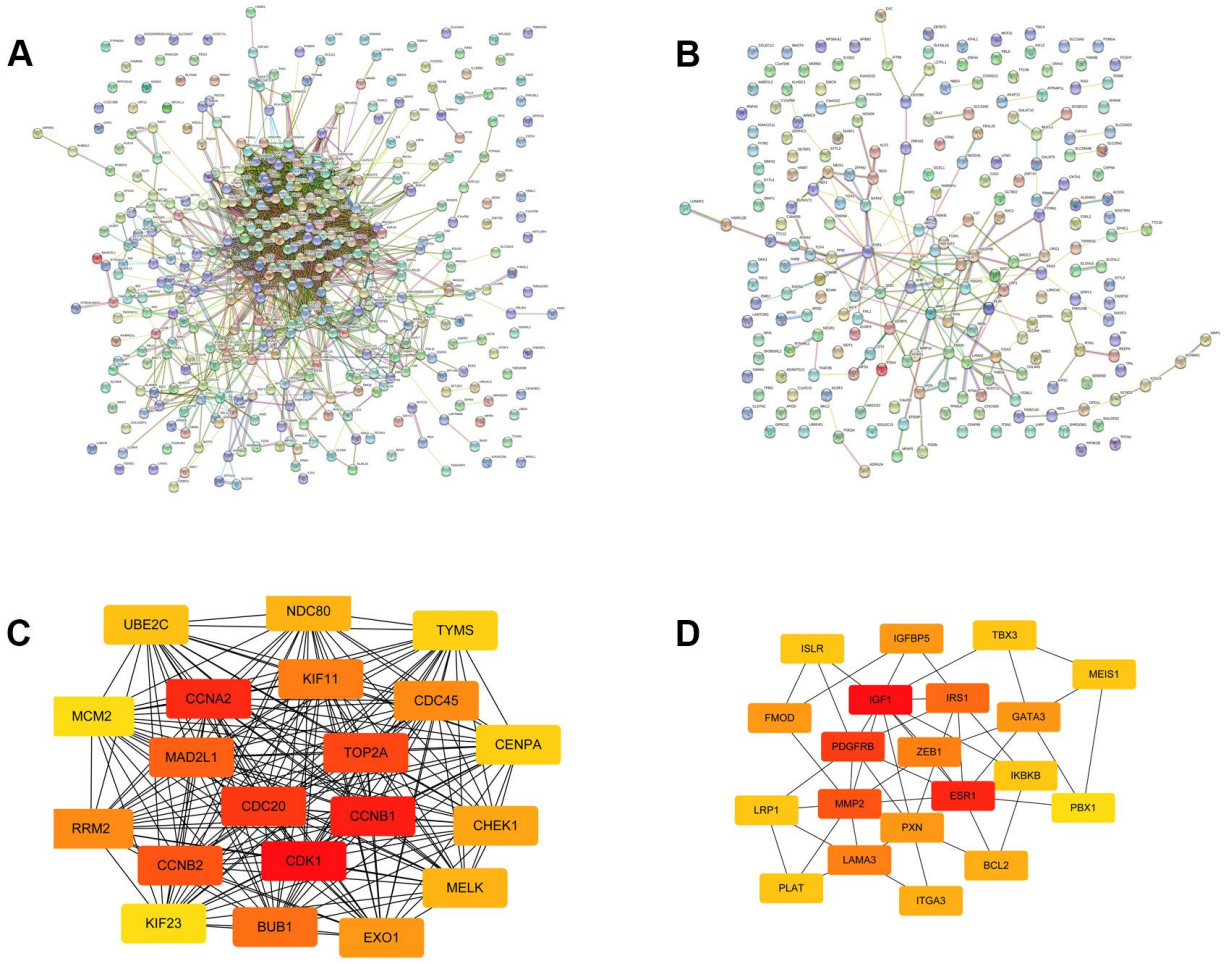
**The top 20 hub genes selected from the PPI networks.** (**A**) The PPI network of the upregulated significant DE-mRNAs. (**B**) The PPI network of the downregulated significant DE-mRNAs. (**C**) The top 20 hub genes of the upregulated significant DE-mRNAs. (**D**) The 20 hub genes of the downregulated significant DE-mRNAs.

To explore the protein interaction networks, we used the STRING database to construct the PPI networks of the identified DE-mRNAs, as shown in Figure 3A, 3B. Based on the node degree, the top 20 hub genes in the downregulated and upregulated DE-mRNAs were identified using Cytoscape software and are listed in Table 1. To visualize better, the interactions of the top 20 hub genes were reconstructed and are presented in Figure 3C, 3D. The top 10 of these 20 hub genes were selected for further analyses.

**Table 1:** The top 20 hub genes in the PPI networks.

**Overexpressed gene**		**Underexpressed gene**	
**Gene symbol**	**Degree**	**Gene symbol**	**Degree**
CDK1	113	ESR1	13
CCNB1	108	IGF1	10
CCNA2	105	PDGFRB	8
CDC20	104	PXN	7
TOP2A	103	ZEB1	6
CCNB2	102	MMP2	6
MAD2L1	99	IRS1	6
BUB1	96	BCL2	5
KIF11	94	PPID	4
RRM2	89	ITGA3	3
CDC45	89	RUNX1T1	3
EXO1	86	NEGR1	3
CHEK1	85	PDGFD	3
MELK	84	MEIS1	3
NDC80	84	PBX1	3
UBE2C	83	FRS2	2
TYMS	82	ALCAM	2
CENPA	82	TOX3	2
KIF23	80	WISP2	2
MCM2	80	RTN1	2

### Analysis of expression and prognosis of hub genes in breast cancer

**Figure 4:**
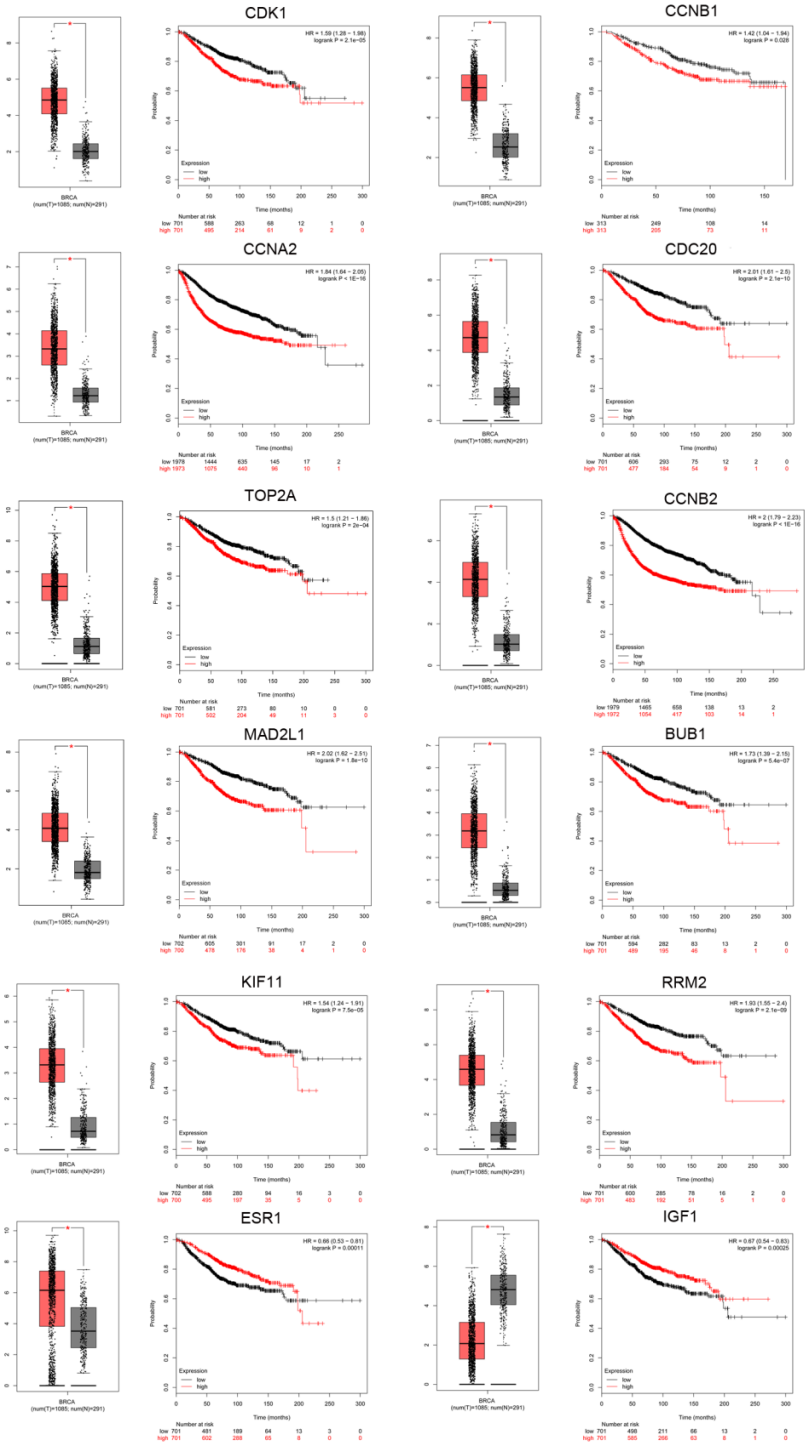
**Screening of key genes in TNBC.** Key genes were identified from the top 10 hub genes of the significant dysregulated DE-mRNAs by merging the prognosis and expression analyses using Kaplan Meier-plotter and GEPIA databases. Expression boxplots and survival curves (overall survival [OS]) of 12 key genes, including 10 upregulated hub genes (CDK1, CCNB1, CCNA2, CDC20, TOP2A, CCNB2, MAD2L1, BUB1, KIF11, and RRM2) and two downregulated hub genes (ESR1 and IGF1) in TNBC are presented.

To further examine the expression of the hub genes in TNBC, the expression of the top 10 upregulated and top 10 downregulated hub genes was analyzed using the GEPIA database. All of the 10 upregulated hub genes (CDK1, CCNB1, CCNA2, CDC20, TOP2A, CCNB2, MAD2L1, BUB1, KIF11, and RRM2) were dramatically upregulated in breast cancer, whereas five of the 10 downregulated hub genes (ESR1, IGF1, PDGFRB, PXN, and ZEB1) were significantly downregulated in breast cancer. The ability of the hub genes to predict prognosis in TNBC was evaluated using the Kaplan-Meier (KM) plotter database. The 10 upregulated hub genes correlated significantly with poor disease outcome. Of the downregulated hub genes, only ESR1 and IGF1 were correlated with favorable prognosis in TNBC. Expression boxplots and survival curves are shown separately in Figure 4. Thus, these 10 overexpressed and two underexpressed hub genes were selected for further investigation. In addition, the expression of the 12 key genes was verified in TNBC samples from The Cancer Genome Atlas (TCGA) (Supplementary Table 2).

### Validation and prediction of upstream key miRNAs

**Figure 5:**
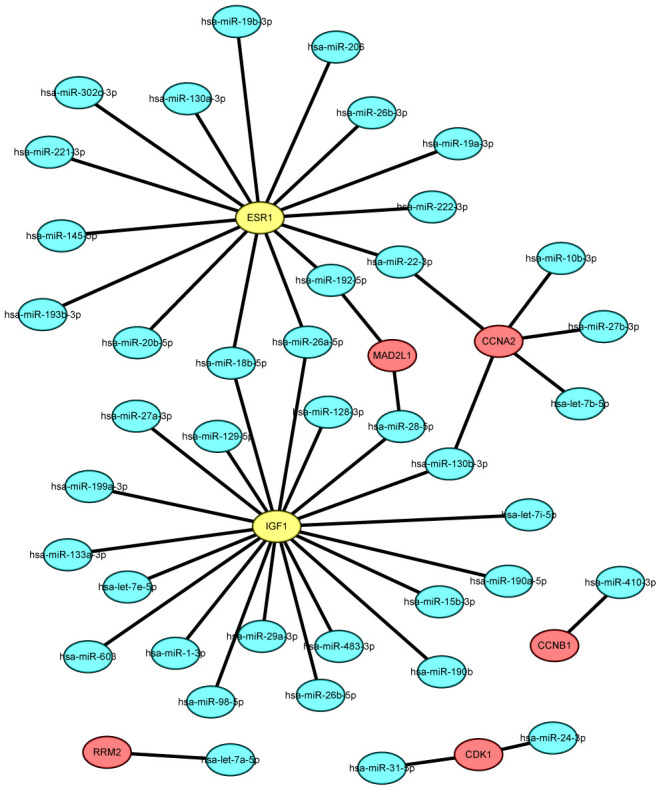
**The mRNA-miRNA network established by Cytoscape software.**

Key miRNAs that regulate the 12 identified hub genes were predicted using miRTarBase. In view of the credibility of the predicted results, only microRNA–target gene interactions proved by reporter assay were selected. As presented in Figure 5 and Supplementary Table 3, 11 miRNAs that could possibly modulate five of the key upregulated genes (CCNA2, MAD2L1, CDK1, RRM2, and CCNB1) and 32 miRNAs that could potentially modulate the two key downregulated genes (ESR1 and IGF1) were identified. Upstream potential miRNAs of five key genes (CDC20, TOP2A, MAD2L1, BUB1, and KIF11) were not included. We then evaluated the expression pattern and prognostic value of the predicted miRNAs in breast cancer patients using the starBase and KM plotter databases according to the inverse regulation between miRNA and mRNA. Consequently, for upregulated hub genes, four miRNAs (hsa-let-7b-5p, hsa-miR-10b-3p, hsa-let-7a-5p, and hsa-miR-410-3p) were not only downregulated but also linked to favorable disease outcomes in patients with breast cancer. For downregulated hub genes, six miRNAs (hsa-miR-19a-3p, hsa-let-7e-5p, hsa-miR-130b-3p, hsa-miR-98-5p, hsa-miR-18b-5p, and hsa-miR-222-3p) were significantly upregulated and correlated with poor prognosis (*P* < 0.05). The expression boxplots and survival curves of the 10 key miRNAs are provided in Supplementary Figures 1, 2, respectively.

### Validation and prediction of upstream key lncRNAs

**Figure 6:**
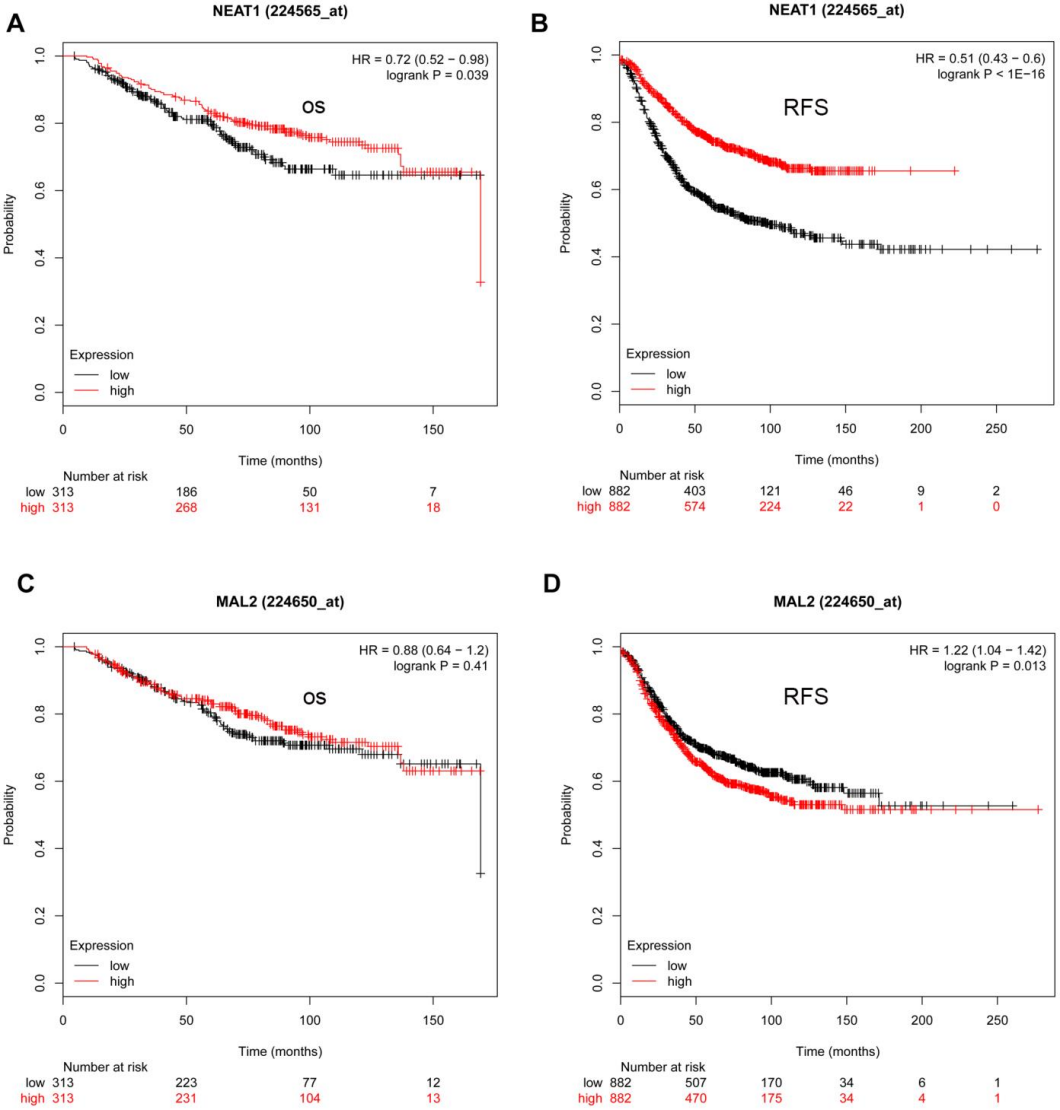
**The prognostic values of NEAT1 and MAL2 in TNBC determined by the Kaplan-Meier plotter.** (**A**) The prognostic value (overall survival [OS]) of NEAT1 in TNBC. (**B**) The prognostic value (relapse-free survival [RFS]) of NEAT1 in TNBC. (**C**) The prognostic value (OS) of MAL2 in TNBC. (**D**) The prognostic value (RFS) of MAL2 in TNBC.

Studies have demonstrated that lncRNAs suppress miRNA expression by acting as miRNA sponges [11–13]. As a result, we predicted the important upstream lncRNAs that could possibly bind to the 10 vital miRNAs (hsa-let-7b-5p, hsa-miR-10b-3p, hsa-let-7a-5p, hsa-miR-410-3p, hsa-miR-19a-3p, hsa-let-7e-5p, hsa-miR-130b-3p, hsa-miR-98-5p, hsa-miR-18b-5p, and hsa-miR-222-3p) using the online miRNet database. In total, 374 lncRNA-miRNA pairs were identified (Supplementary Table 4). According to the ceRNA hypothesis, expression of these lncRNAs was evaluated using the GEPIA database. When compared with normal controls, nine lncRNAs (AC018766.4, CROCCP2, CTD-3092A11.2, LINC00342, RP11-553L6.5, XXbac-B461K10.4, NEAT1, RP11-228B15.4, and RP11-311C24.1) that target to upregulated miRNAs showed lower expression in breast cancer, whereas five lncRNAs (HOTAIR, LINC00467, RECQL4, LINC00665, and MAL2) that target to downregulated miRNAs were significantly upregulated in breast cancer (Supplementary Figure 3). Subsequent survival analysis using the KM plotter database revealed that patients with low NEAT1 expression and high MAL2 expression had an unfavorable prognosis. Thus, NEAT1 and MAL2 were recognized as key lncRNAs (Figure 6).

### Establishment of the mRNA-miRNA-lncRNA regulatory prognostic network in TNBC

**Figure 7:**
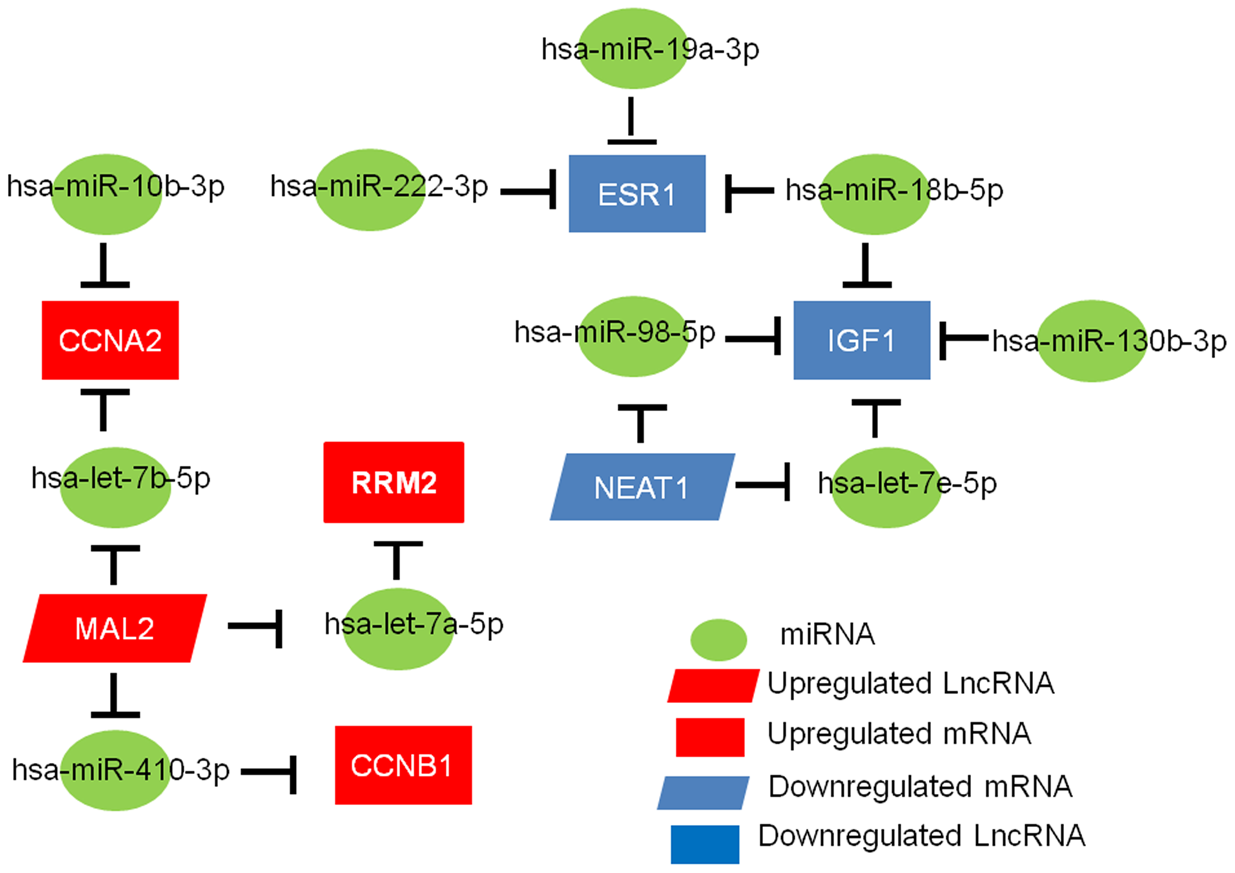
**The mRNA-miRNA-lncRNA competing endogenous RNA (ceRNA) network related to the prognosis of TNBC.**

We applied bioinformatics to develop a key lncRNA-miRNA-mRNA ceRNA network in TNBC. As depicted in Figure 7, the network includes 11 miRNA-mRNA pairs (miR-19a-3p-ESR1, miR-18b-5p-ESR1, miR-222-3p-ESR1, let-7e-5p-IGF1, miR-130b-3p-IGF1, miR-98-5p-IGF1, miR-18b-5p-IGF1, let-7b-5p-CCNA2, miR-10b-3p-CCNA2, let-7a-5p-RRM2, and miR-410-3p-CCNB1), five miRNA-lncRNA pairs (let-7e-5p-NEAT1, miR-98-5p-NEAT1, let-7a-5p-MAL2, miR-410-3p-MAL2, and let-7b-5p-MAL2), and four mRNA-lncRNA pairs (IGF1-NEAT1, RRM2-MAL2, CCNB1-MAL2, and CCNA2-MAL2). Based on the ceRNA hypothesis, lncRNA works as a ceRNA to compete for shared miRNA and sequester miRNA away from mRNA. LncRNA has an inverse co-expression relationship with miRNA but a positive co-expression relationship with mRNA. Therefore, the correlations between mRNA-lncRNA, miRNA-lncRNA, and mRNA-miRNA pairs in the constructed network were assessed using the starBase database, and results are shown in Table 2. Except for one miRNA-lncRNA pair (let-7e-5p-NEAT1), the other pairs were all fitted with the ceRNA mechanism. By considering the three levels, a novel mRNA-miRNA-lncRNA triple subnetwork, including four mRNA-miRNA-lncRNA axes (IGF1-miR-98-5p-NEAT1, RRM2-let-7a-5p-MAL2, CCNB1-miR-410-3p-MAL2, and CCNA2-let-7b-5p-MAL2), was ultimately constructed and possessed significant prognostic value in TNBC.

**Table 2:** The correlation between mRNA, miRNA, and lncRNA according to the starBase database.

**miRNA**	**mRNA**	**R**	***P* value**
hsa-miR-19a-3p	ESR1	–0.426	5.47e-49
hsa-miR-18b-5p	ESR1	–0.276	1.85e-20
hsa-miR-222-3p	ESR1	–0.306	6.71e-25
hsa-let-7e-5p	IGF1	–0.058	5.80e-2
hsa-miR-130b-3p	IGF1	–0.304	1.25e-24
hsa-miR-98-5p	IGF1	–0.182	1.58e-9
hsa-miR-18b-5p	IGF1	–0.092	2.45e-3
hsa-let-7b-5p	CCNA2	–0.239	1.62e-15
hsa-miR-10b-3p	CCNA2	–0.301	3.88e-24
hsa-let-7a-5p	RRM2	–0.306	5.49e-25
hsa-miR-410-3p	CCNB1	–0.189	3.49e-10
**miRNA**	**lncRNA**	**R**	***P* value**
hsa-let-7e-5p	NEAT1	0.057	5.85e-02
hsa-miR-98-5p	NEAT1	–0.198	5.24e-11
hsa-let-7a-5p	MAL2	–0.066	2.96e-02
hsa-miR-410-3p	MAL2	–0.144	2.01e-06
hsa-let-7b-5p	MAL2	–0.007	8.28e-1
**mRNA**	**lncRNA**	**R**	***P* value**
IGF1	NEAT1	0.142	2.04e-06
RRM2	MAL2	0.139	3.77e-06
CCNB1	MAL2	0.223	7.46e-14
CCNA2	MAL2	0.207	4.11e-12

## DISCUSSION

Breast cancer is associated with high mortality in women, and incidence is increasing worldwide. TNBC is the most fatal subtype of breast cancer. In addition, the therapeutic options for TNBC are limited.

Chemotherapy remains the primary therapy for advanced TNBC. Because it is believed that development of TNBC is regulated by sophisticated signaling networks, revealing the specific molecular mechanism of TNBC could result in development of effective treatments and novel prognostic biomarkers.

Recently, a novel mechanistic function of lncRNAs, known as ceRNAs, has been reported. lncRNAs sponge miRNAs to regulate their expression, thereby contributing to various pathological processes, including the development of cancer [12–14]. For example, Zhang et al. reported that a downregulated lncRNA, MT1JP, affected the progression of gastric cancer by competitively binding to miR-92a-3p and regulating FBXW7 expression [15]. A study by Wang et al. determined that the lncRNA HOXD-AS1 can bind to miR-130a-3p and inhibit SOX4 degradation, thus activating MMP2 and EZH2 expression and promoting the metastasis of hepatocellular carcinoma [16]. Wu et al. observed that, in papillary thyroid carcinoma, the lncRNA SNHG15 can serve as a ceRNA and thus modulate YAP1-Hippo signal transduction by sponging miR-200a-3p [17]. Luan et al. found that there is crosstalk between the lncRNA XLOC_006390 and miR-338-3p and miR-331-3p expression, which aggravates cervical cancer [18]. Studies have also suggested that ceRNA influences the pathogenesis of breast cancer. Zhao et al. suggested that the lncRNA TUSC8 may affect epithelial-mesenchymal transition (EMT)-associated protein levels by functioning as a ceRNA of myosin regulatory light chain interacting protein (MYLIP) as it binds miR-190b-5p, leading to the suppression of breast cancer progression [19]. Lu et al. found that lncARAP1-AS promotes tumorigenesis by enhancing the proliferative and migratory abilities of breast cancer cells by modulating the miR-2110/HDAC2/PLIN1 axis [20]. However, there are different breast cancer subtypes, including HER2-positive breast cancer, luminal A and B breast cancer, and TNBC, and comprehensive studies on the ceRNA networks in each breast cancer subtype are limited, particularly regarding the identification of their specific molecular mechanisms.

In this study, we identified 1482 DE-mRNAs in TNBC and then constructed a unique lncRNA-miRNA-mRNA network based on the ceRNA hypothesis. To our knowledge, we are the first to identify the specific ceRNA network involved in TNBC using a stepwise reverse prediction from mRNA to lncRNA, instead of using the lncRNA-miRNA-mRNA pattern. In addition, each component in this network is significantly associated with breast cancer prognosis, providing potential therapeutic targets and prognostic biomarkers.

First, we screened for significantly dysregulated mRNAs in TNBC tissues compared with normal control tissue and non-TNBC breast cancer tissue in two GEO data sets (GSE36259 and GSE42568). A total of 860 upregulated mRNAs and 622 downregulated mRNAs were selected as specific DE-mRNAs in TNBC. GO analysis [21] indicated that these DE-mRNAs are significantly enriched in some factors of cell division, proliferation, and response, such as centromere complex assembly [22], mitotic sister chromatid segregation and DNA replication [23], insulin-like growth factor receptor signaling pathway [24], and transmembrane transporter activity [25, 26]. Subsequently, KEGG pathway analysis indicated that these dysregulated DE-mRNAs were remarkably enriched in damage repair, cell death, and cancer-related pathways, including the p53 signaling pathway [27, 28], mismatch repair [29, 30], cellular senescence [31], ferroptosis [32, 33], prostate cancer, and small-cell lung cancer. Therefore, these DE-mRNAs identified through the intersection of two GEO data sets were found to be closely associated with the pathogenesis of TNBC.

To explore the potential association among these identified DE-mRNAs, two PPI networks were established via the STRING database and exhibited a variety of interactions among the DE-mRNAs, especially in the upregulated group. Genes that have a greater node degree in the PPI network typically play vital roles. Thus, the hub genes in the two PPI networks we screened had node degree calculated by Cytoscape software. In addition, the top 10 overexpressed and underexpressed hub genes were chosen for survival and expression analyses to identify key genes in TNBC. All 10 upregulated hub genes (CDK1, CCNB1, CCNA2, CDC20, TOP2A, CCNB2, MAD2L1, BUB1, KIF11, and RRM2) and two downregulated hub genes (ESR1 and IGF1) were found to affect progression of TNBC (ie, they are key genes). Interestingly, based on previous reports, in addition to breast cancer, most of these hub genes are also related to other cancers. For example, in pancreatic ductal adenocarcinoma, overexpression of CDK1 indicates poor prognosis [34], and CCNA2 is considered a possible biomarker for progression (eg, growth and apoptosis) of colorectal cancer [35]. Evidence indicates that CCNB1 can predict the prognosis of ER-positive breast cancer, as well as the efficacy of hormonal therapy [36]. ESR1 mutation results in acquired endocrine resistance in breast cancer [37].

We initially predicted the miRNAs of the hub genes as described previously based on the ceRNA mechanism. After the expression and survival analyses, we identified 10 key miRNAs, among which six upregulated miRNAs (hsa-let-7e-5p, hsa-miR-19a-3p, hsa-miR-130b-3p, hsa-miR-18b-5p, hsa-miR-98-5p, and hsa-miR-222-3p) were significantly associated with poor prognosis and four downregulated miRNAs (hsa-let-7b-5p, hsa-miR-10b-3p, hsa-let-7a-5p, and hsa-miR-410-3p) were associated with better prognosis in breast cancer. Aberrant expression patterns of these key miRNAs contribute to the progression and prognosis of many cancers. For instance, the upregulation of MiR-98-5p enhances the progression of non-small-cell lung cancer, as well as breast cancer [38, 39]. In addition, the upregulation of miR-18a-5p enhances the ability of lung adenocarcinoma cells to proliferate via the miR-18b-5p/VMA21 axis [40]. The miR-18b-5p/DOCK4 axis inhibits the EMT and migratory capacity of breast cancer cells [41]. Let-7b-5p suppresses the motility and proliferation of squamous cell carcinoma cells [42]. miR-10b-3p expression facilitates the pathogenesis of liver cancer by interacting with CMTM5 [43]. Yang et al. conducted bioinformatics analysis and reported that miR-10b-3p can act as a prognostic biomarker in colorectal cancer [44]. MiR-130b-5p was also found to contribute to the occurrence of pancreatic ductal adenocarcinoma [45].

In our study, we conducted further analysis to identify upstream lncRNAs for the key miRNAs. By combining survival analysis and expression validation, only two lncRNAs (NEAT1 and MAL2) were selected as the key lncRNAs. Overexpression of NEAT1 has been observed in various tumors and has been associated with tumorigenesis and poor prognosis [46–48]. Li et al. implicated the ERα-NEAT1-FOXN3/NEAT1/SIN3A-GATA3 axis in the metastasis of breast cancer [49]. The oncogenic effects of NEAT1 have been reported to be influenced by the CDC5L-AGRN transcriptional regulation circuit in prostate cancer [50]. Some studies have shown that MAL2 functions as an oncogene in various malignant tumors [51]. Bhandari et al. demonstrated that MAL2 is elevated in breast cancer tissues and that the upregulation of MAL2 was related to the lowest OS rate in the TCGA cohort, suggesting that MAL2 could be an oncogene for breast cancer [52].

This study has some limitations. After identification of candidate DE-mRNAs in TNBC tissues, the screening of key mRNAs, miRNAs, and lncRNAs was performed according to their expression and prognostic values among the whole breast cancer group instead of the TNBC subgroup. Expressions of 12 key genes was further confirmed in TNBC samples from TCGA. The limited TNBC samples in both the GEO and TCGA data sets limited our ability to perform statistically significant analysis of the expression and prognosis of key genes in TNBC. Therefore, we did not stratify the tumor samples and predicted them based on all breast cancers. In addition, both the expression and prognostic values were considered during screening, which could have resulted in our missing some valuable molecules.

In conclusion, we successfully established a novel mRNA-miRNA-lncRNA regulatory network in TNBC using comprehensive bioinformatics analysis. Each component of the network has significant prognostic predictive value for TNBC. Co-expression analysis for all of the RNA pairs in the network revealed that four mRNA-miRNA-lncRNA axes (IGF1-miR-98-5p-NEAT1, RRM2-let-7a-5p-MAL2, CCNB1-miR-410-3p-MAL2, and CCNA2-let-7b-5p-MAL2) act as key ceRNA subnetworks and can be used to predict prognosis or serve as treatment targets in TNBC. Experimental validation of the network should be conducted in future trials.

## MATERIALS AND METHODS

### Gene expression profile data

To compare genome-wide gene expression profiles among TNBC tissues, non-TNBC tissues, and normal tissues, data sets from the GEO (https://www.ncbi.nlm.nih.gov/geo/) database were searched according to our selection criteria. Data sets containing the mRNA expression profiling on triple-negative primary breast cancer (TNPBC) tissues, non-TNPBC tissues such as luminal and HER2-positive breast cancer, and normal tissues were exclusively enrolled. Each of the three groups contains no less than 10 samples. Details of the data sets, including the summary and overall design, were further evaluated. Finally, the GSE45827 (including 41 TNBC samples, 89 non-TNBC samples, and 11 normal tissue samples) and GSE65194 (including 55 TNBC samples, 98 non-TNBC samples, and 11 normal tissue samples) data sets were chosen for subsequent study. To enhance the reliability of screening results, the TNBC TCGA data sets were added as validation data sets.

### Differential expression screening

Upregulated or downregulated DE-mRNAs in the two selected data sets between TNPBC tissues and the other two groups (non-TNPBC tissues and normal tissues) were assessed using the online analytic tool GEO2R (https://www.ncbi.nlm.nih.gov/geo/), and the cutoff criteria were set as |log2FC| > 1 and *P* value < 0.05 when analyzing differential expression. Next, Venn diagrams were constructed using VENNY 2.1.0 (http://bioinfogp.cnb.csic.es/tools/venny/index.html). In all four differential expression analyses, the commonly dysregulated DE-mRNAs were redefined as the significant DE-mRNAs and were chosen for further study.

The clinical and RNA sequencing raw data from 113 normal samples and 1109 breast cancer tumor samples were obtained from TCGA database, and 149 TNBC tissues and 12 paired paracancer tissues were selected. The raw data were first standardized using the method of log_2_(*x* + 1). Then, the normalized processing of data was conducted using the normalize Between Array function from R package LIMMA. Subsequently, the LIMMA package in R (version 3.4.1) was used to identify DE-mRNAs between TNBC tissues and normal breast tissues in TCGA data sets, and the cutoff criteria were set as adjusted *P* < 0.05 and |logFC| >1.

### Functional enrichment analysis

For the commonly identified DE-mRNAs, GO functional annotation and analysis of biological pathways based on the KEGG pathway database were conducted using Enrichr, a comprehensive gene set enrichment analysis (http://amp.pharm.mssm.edu/Enrichr/). The top 10 enriched GO items and pathways were visualized and downloaded directly from the webpage.

### Construction of a PPI network and screening for hub genes

To assess the interactions between DE-mRNAs, the PPI interaction networks were explored using the Search Tool for the Retrieval of Interacting Genes (STRING) database (https://string-db.org/) [53]. The interactors with a combined confidence score ≥ 0.4 were used to generate the PPI network, and the comprehensive interaction pair information of these differentially expressed genes was downloaded from STRING. Based on the connection degree, the hub genes in the PPI networks were subsequently selected using the Cytoscape plugin, CytoHubba [54]. Finally, the top 20 genes of the common DE-mRNAs were found in Cytoscape (Version 3.6.1), identified as hub genes, and ranked by node degree.

### Gene expression analysis

The Gene Expression Profiling Interactive Analysis (GEPIA; http://gepia.cancer-pku.cn/detail.php) database contains RNA sequencing expression data from 8587 normal samples and 9736 tumors obtained from Genotype-Tissue Expression data set projects and TCGA [55]. We used the GEPIA database, which contains 1085 breast cancer samples and 291 normal controls, to identity DE-mRNAs and lncRNAs in TNBC. *P* < 0.05 was chosen as the cutoff value for statistical significance.

### Survival analysis

Prognostic values of mRNAs, lncRNAs, and miRNAs in TNBC were analyzed using the Kaplan-Meier (KM) plotter database (http://kmplot.com) [56], a commonly used website tool to simultaneously integrate gene expression and clinical data retrieved from the GEO, EGA, and TCGA. Subsequently, the potential of these biomarkers to determine the prognosis of cancer was examined. First, the mRNAs, lncRNAs, and miRNAs were entered into the database. Next, KM graphs were generated using the KM plotter database. The log-rank *P* value and hazard ratio (HR) with 95% confidence intervals were calculated. Log-rank *P* < 0.05 was considered statistically significant.

### Prediction of miRNA

To predict the upstream miRNAs of key DE-mRNAs, we obtained miRNA-mRNA interactions from miRTarBase (http://mirtarbase.mbc.nctu.edu.tw) in which the interactions between miRNA targets were confirmed using reporter assay, quantitative polymerase chain reaction, next-generation sequencing, western blot, and microarray experiments [57]. To identify more reliable candidate results, only the miRNA-target interactions verified by reporter assay were collected for further study. Then, the expression of the target miRNA in breast cancer and normal tissues was exported using starBase v3.0, an online platform to investigate the differential expression analysis of miRNAs data from the TCGA [58]. Statistical significance was set at *P* < 0.05.

### Prediction of lncRNA

The miRNet database, which incorporates data from 11 integrated microRNA databases, was used to determine the upstream lncRNAs of miRNA [59]. Selection criteria were ‘‘target type-lncRNAs” and “Organism-H.sapies.” Expression of the candidate target lncRNAs was further assessed using the GEPIA database.

### Correlation analysis

After miRNA-mRNA, mRNA-lncRNA, and miRNA-lncRNA pairs were obtained, the starBase database (http://starbase.sysu.edu.cn/), which contains ncRNA data sets, was used to investigate the correlations [58] between the pairs in invasive breast carcinoma. *P* < 0.05 was set as the cutoff for statistical significance.

### Data availability

Additional data for this work can be obtained from the corresponding author upon request.

## Supplementary Material

Supplementary Figures

Supplementary Table 1

Supplementary Tables 2 and 3

Supplementary Table 4
