# Supplementary material for: Construction of an mRNA-miRNA-lncRNA network prognostic for triple-negative breast cancer
Source: Aging (Albany NY). 2021 Jan 3;13(1):1153–75. doi: 10.18632/aging.202254 (PMC7835059; doi:10.18632/aging.202254)
Supplement: Supplementary Figures [file aging-13-202254-s001.pdf]

SUPPLEMENTARY FIGURES

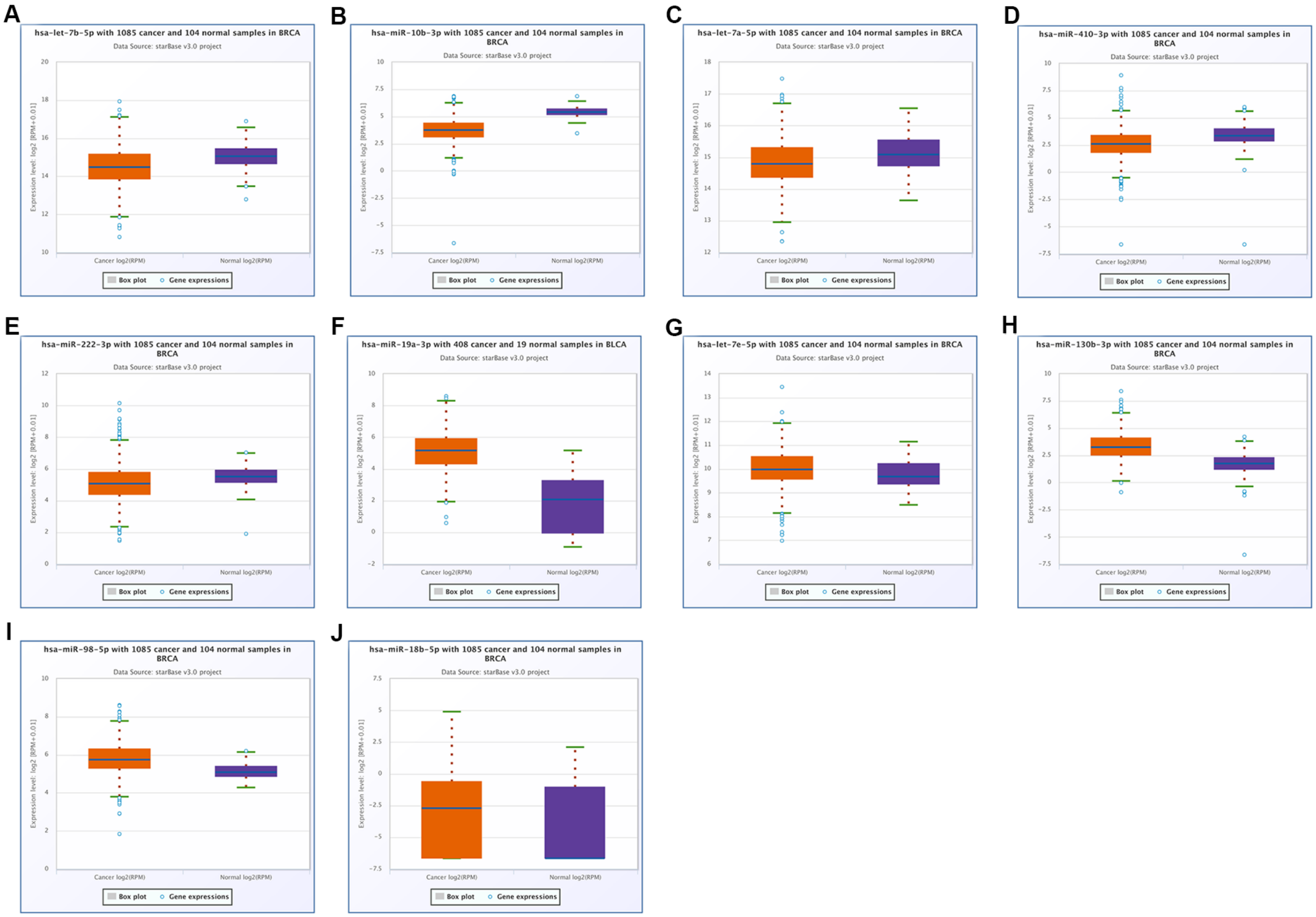

Supplementary Figure 1. The expression of 10 key miRNAs in breast cancer. (**A**) hsa-let-7b-5p. (**B**) hsa-miR-10b-3p. (**C**) hsa-let-7a-5p. (**D**) hsa-miR-410-3p. (**E**) hsa-miR-222-3p. (**F**) hsa-miR-19a-3p. (**G**) hsa-let-7e-3p. (**H**) hsa-miR-130b-3p. (**I**) hsa-miR-98-5p. (**J**) hsa-miR-18-5p.

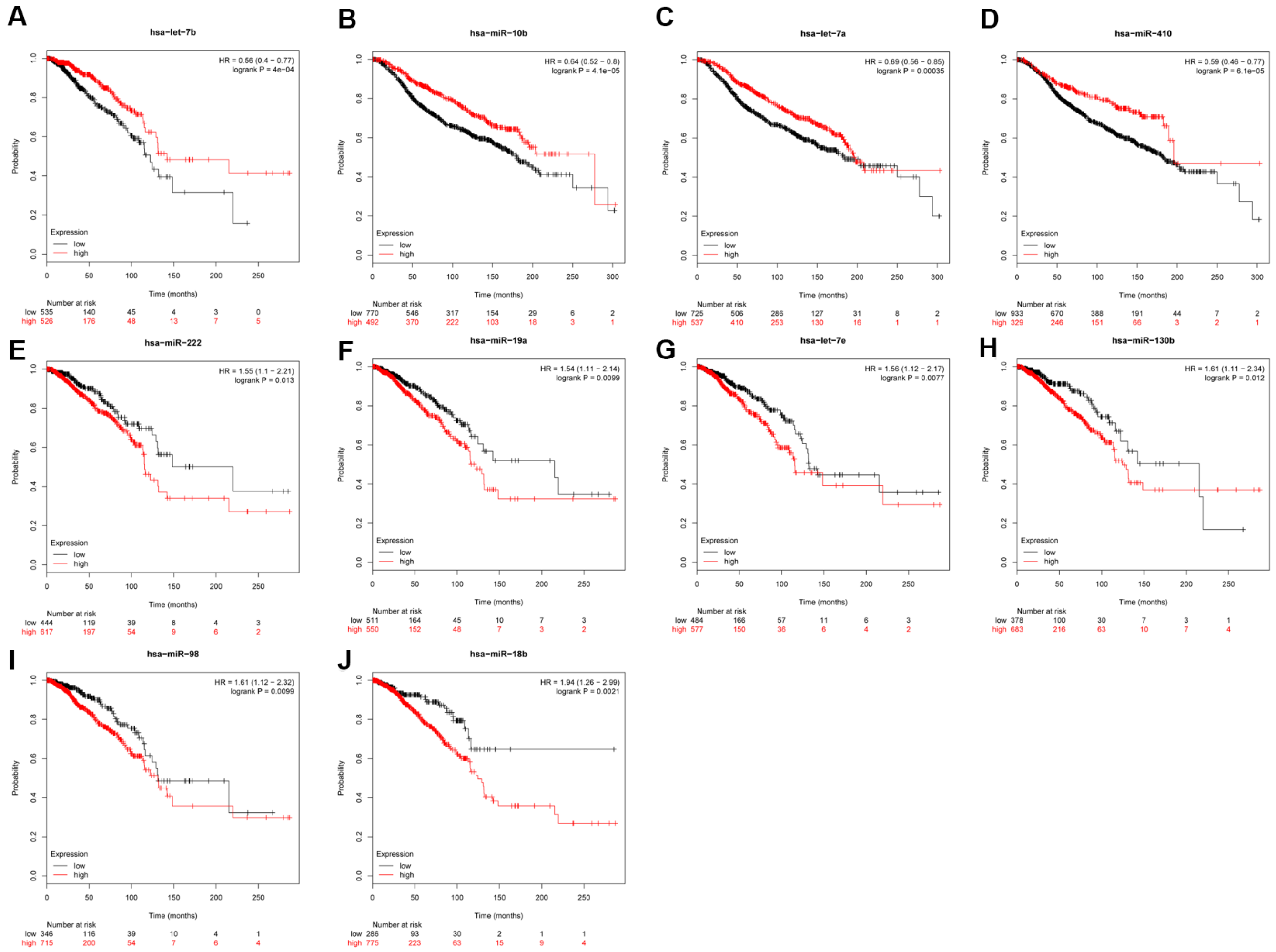

Supplementary Figure 2. The prognostic value (overall survival [OS]) of 10 key miRNAs in breast cancer. (**A**) hsa-let-7b-5p. (**B**) hsa-miR-10b-3p. (**C**) hsa-let-7a-5p. (**D**) hsa-miR-410-3p. (**E**) hsa-miR-222-3p. (**F**) hsa-miR-19a-3p. (**G**) hsa-let-7e-3p. (**H**) hsa-miR-130b-3p. (**I**) hsa-miR-98-5p. (**J**) hsa-miR-18-5p.

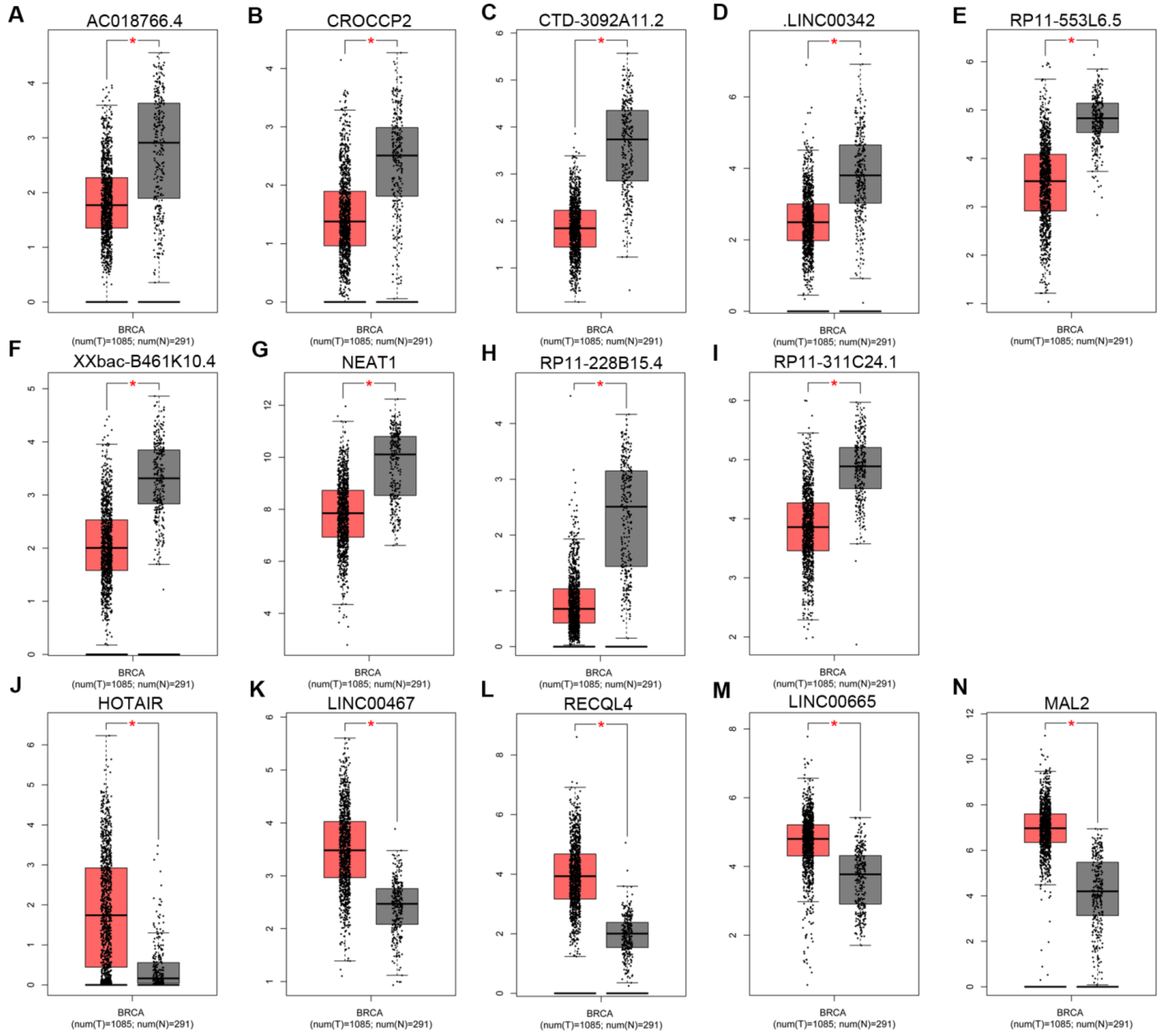

Supplementary Figure 3. The expression of key lncRNAs in breast cancer. (**A**) AC018766.4. (**B**) CROCCP2. (**C**) CTD-309A11.2. (**D**) LINC00342. (**E**) RP11-553L6.5. (**F**) XXbac-B461K10.4. (**G**) NEAT1. (**H**) RP11-228B15.4. (**I**) RP11-311C24.1. (**J**) HOTAIR. (**K**) LINC00467. (**L**) RECQL4. (**M**) LINC00665. (**N**) MAL2.
