# Supplementary material for: Construction of an mRNA-miRNA-lncRNA network prognostic for triple-negative breast cancer
Source: Aging (Albany NY). 2021 Jan 3;13(1):1153–75. doi: 10.18632/aging.202254 (PMC7835059; doi:10.18632/aging.202254)
Supplement: Supplementary Table 1 [file aging-13-202254-s002.docx]

**Supplementary Table 1. Common differentially expressed mRNAs in the GSE45827 and GSE65194 datasets.**

| Upregulated gene | Downregulated gene |
| --- | --- |
| Gene symbol | Gene symbol |
| LOC100131262 | C9orf152 |
| PFKP | MMP2 |
| KCNG1 | KCNMA1 |
| IRAK1 | APBB2 |
| 3-Sep | SLC25A3 |
| RDH10 | IGF1 |
| GPSM2 | FAM214A |
| SLC2A5 | AHSA2 |
| CCNB1 | TMC4 |
| CCDC28B | LINC01279 |
| LOC100129518///SOD2 | SIDT1 |
| LYAR | HMGCS2 |
| PIR | FAM134B |
| SRPK1 | WISP2 |
| DHRS11 | MITF |
| CMPK2 | C12orf66 |
| RUNX3 | RTN1 |
| USP6NL | REEP6 |
| LMNB2 | COL4A5 |
| ATL2 | ZEB1 |
| PDCD5 | MSRB3 |
| LOC284926 | NOSTRIN |
| RBM17 | EVC |
| SEL1L3 | SUSD2 |
| ARHGEF9 | NFIA |
| CD38 | FRY |
| CHRAC1 | ELOVL5 |
| PGBD5 | ITGA3 |
| CCNE1 | HNMT |
| FOXM1 | GLT8D2 |
| ORM1 | CNN1 |
| HSPD1 | SLC25A46 |
| CDK1 | SHROOM1 |
| DESI1 | C16orf45 |
| OR7E37P | GHR |
| PREP | FITM2 |
| NFE4 | VWA5A |
| FAXC | DUSP4 |
| ATP11C | PODN |
| CLCN4 | PSMG4 |
| CXorf56 | FRS2 |
| EN1 | KIAA1211L |
| EMC8 | N4BP2L2 |
| CNOT1 | SLC16A5 |
| CDC42EP3 | HSPB2-C11orf52///C11orf52///HSPB2 |
| LOC202181 | TPCN1 |
| TTLL4 | STARD13 |
| DAPP1 | PPM1A |
| NFIL3 | PCDH7 |
| CENPU | BCL2 |
| MRPL15 | MMP16 |
| RPUSD3 | PGGHG |
| KLHL7 | MYO15B |
| TDRKH | LRRC4C |
| GNB4 | ALCAM |
| AGTPBP1 | KIAA1324 |
| KIF14 | ORAI3 |
| MAD2L1 | C1orf168 |
| MUC15 | BCAM |
| SH2D2A | C14orf132 |
| LMO4 | TOX3 |
| MAGOHB | THRB |
| C1orf112 | ADRA2A |
| MICALL1 | EFHC1 |
| UBD///GABBR1 | IRS1 |
| LOC100131270///LOC100129029 | PIP4K2B |
| APOBEC3A_B///APOBEC3A | NUDT12 |
| SOX9 | CLSTN2 |
| CRABP1 | KDM4B |
| KIAA0226L | MFAP5 |
| RDX | CST3 |
| NDRG2 | TFAP2B |
| ADCY3 | NEBL |
| GRHL1 | LINC00993 |
| CENPL | TPBG |
| KIF4A | LZTFL1 |
| POLR3G | TCF4 |
| TANK | LONRF2 |
| RCAN1 | GPRC5C |
| BICD1 | PBLD |
| CA9 | AHNAK |
| GLIPR2 | FHL1 |
| SEPHS1 | RTN4RL1 |
| EIF5A | PBX1 |
| FAM49A | ZNF747 |
| TCF19 | ACSF2 |
| MCM10 | DUBR |
| CBS | SYTL5 |
| FAM72A///FAM72D///FAM72B///FAM72C | OLFML2A |
| TYMS | HID1 |
| ZNF750 | LOC101930404///SNORD116-28///SNORD115-26///SNORD115-13///SNORD115-7///SNORD116-22///SNORD116-4///PWARSN///SNORD107///IPW |
| MCM5 | NUAK1 |
| MELK | ZFPM2 |
| IL15RA | SYTL4 |
| PTP4A3 | ATP6AP1L |
| PARVB | PDGFRB |
| NDC80 | ALDH6A1 |
| SGO1 | OMD |
| MYC | ITSN1 |
| PRKY///PRKX | CMYA5 |
| OIP5 | PXN |
| CCNA2 | DIXDC1 |
| E2F7 | CADPS2 |
| GAL | MEIS1 |
| THEMIS2 | NEDD4L |
| COCH | PDGFD |
| PSRC1 | FST |
| HRASLS | FAM3B |
| MMP1 | KCND3 |
| CBX2 | TRIL |
| TRIM16 | KLF2 |
| STMN1 | TTC12 |
| TRIM47 | FBXL20 |
| CKS2 | NTN4 |
| DEPDC1 | ZDHHC11B///ZDHHC11 |
| KIF23 | LAMTOR3 |
| GNLY | C1orf115 |
| ZNF165 | LOC100507577///LONP2 |
| NDE1 | SH3BGRL2 |
| URB2 | NEGR1 |
| YBX1 | MAP1A |
| NCK1-AS1 | EME2 |
| IDO1 | PPID |
| OGFRL1 | RUNX1T1 |
| ATP11B | LOC101930404///SNORD116-28///SNORD115-26///SNORD115-13///SNORD115-7///SNORD116-22///SNORD116-4///PWARSN///SNORD107///SNRPN///IPW |
| ASH1L-AS1 | ESR1 |
| BATF2 | KIAA0232 |
| CCL18 | CHCHD5 |
| LMNB1 | MIR29C///MIR29B2 |
| FAM83B | KIAA0485 |
| KRTCAP3 | FMOD |
| CLIC4 | LRIG1 |
| SAMHD1 | RNF43 |
| PAX6 | CRAT |
| TAP2 | SETBP1 |
| KLHL15 | AREG |
| SNX5 | ZBTB7C |
| GPR161 | MAST4 |
| LOC101930416///LOC101929792///LOC100996724///PDE4DIP | LAMA3 |
| CCNB2 | SCGB1D2 |
| PRC1 | LFNG |
| LOC100509457///HLA-DQA1 | GPD1L |
| CDT1 | TSPAN31 |
| LRP8 | KIF5C |
| CENPW | PDK4 |
| LOC101928076 | CA12 |
| NFE2L3 | LRRFIP1 |
| ELAC1 | IGFBP5 |
| MPP6 | COLEC12 |
| KRT16 | GALNT10 |
| DDIAS | TRIM6 |
| TMEM39B | RPS6KA2 |
| CMAS | KLHDC1 |
| MOCOS | EFEMP1 |
| TNFRSF21 | WFS1 |
| SLC6A8 | SLC22A5 |
| CCNE2 | SERHL2///SERHL |
| CDC45 | SMOC2 |
| PPP1CB | NR2F2///NR2F1 |
| CHEK2 | CAPN8 |
| MCM2 | MCF2L |
| TMEM200C | ACSS3 |
| MCM4 | CFAP70 |
| PRIM2B///PRIM2 | SHC2 |
| MIR155///MIR155HG | EMCN |
| ZBED4 | HOXD8 |
| ESYT2 | OCEL1 |
| SLAMF7 | SIGLEC15 |
| GPR84 | ZNF44 |
| LRP12 | F2RL2 |
| ODC1 | F13A1 |
| ADORA2B | GATA3 |
| POLH | SLC22A23 |
| NAB1 | OGN |
| PEG3 | INS-IGF2///IGF2 |
| CXCL10 | CEP290 |
| CKAP2L | TTC36 |
| HERC5 | ISLR |
| ARG2 | DKK3 |
| CABYR | LRP1 |
| TOMM5 | SPRY1 |
| CDCA2 | SERPINI1 |
| LOC729680 | PLAT |
| NCK1 | ITGBL1 |
| TRAF3IP3 | KIF12 |
| NPM3 | NBEA |
| RARRES1 | ZDHHC3 |
| ITCH | GALNT6 |
| RPP25 | IKBKB |
| EXO1 | ZMAT1 |
| CCDC71L | ADAMTS15 |
| IGHV3-23///IGHV4-31///IGHM///IGHG4///IGHG3///IGHG1///IGHD///IGHA2///IGHA1///IGH | THBS4 |
| TRMU///GTSE1 | TRIM66 |
| TET1 | SRPX2 |
| PRRT3-AS1 | CFAP69 |
| CENPI | HOXB-AS1 |
| MLKL | C4orf32 |
| OTULIN | ARMC9 |
| CFDP1 | CNTN1 |
| CENPK | MUCL1 |
| MARCO | AKAP12 |
| ASF1A | XIST |
| UPF3B | TMC5 |
| HLA-DOB | HSPA12B |
| POLD1 | APOD |
| SFT2D2 | PDE5A |
| DEK | ELOVL2 |
| LY6D | PTPRG |
| JPH1 | CRIPAK |
| GBP5 | RAI2 |
| MCM3AP-AS1 | SYTL2 |
| STX6 | PWAR6 |
| PDK3 | MKL2 |
| UGT8 | TBX3 |
| RFC5 | MGC24103 |
| HORMAD1 | ZNF423 |
| AKT3 | ARSG |
| LAPTM4B | SEMA6D |
| NEK2 | RAD50 |
| TMEM206 | KCNJ3 |
| RBMS1 | RALGPS2 |
| PNP | LOC388692 |
| FANCD2 | HTRA1 |
| LBR | IFT88 |
| CDC25A | LHFP |
| BAIAP2L1 | NME5 |
| ESPL1 | ARSD |
| B3GNT5 | MZF1 |
| C1orf106 | ADIRF |
| LPP | HEATR6 |
| BID | PGR |
| MIR146A | SRPX |
| KLK6 | FBXL7 |
| TAF4B | SEC14L2 |
| MALT1 | ZFYVE16 |
| TUBGCP3 | POTEM |
| DEPDC1B | SYBU |
| CSAG2///CSAG3 | SGCD |
| SLC25A37 | SEC62 |
| TFDP2 | FLRT3 |
| HCP5 | TP53TG1 |
| FOXQ1 | ERO1B |
| ORM2///ORM1 | STS |
| LOC100509445///LOC728715///OVOS///OVOS2 | KANK2 |
| TBC1D31 | NAV3 |
| GINS3 | LAMB2 |
| ST6GALNAC5 | NBL1 |
| RPP40 | APH1B |
| YOD1 | OLFML3 |
| DCBLD2 | SBSPON |
| DPH3 | PIK3R1 |
| PAFAH1B1 | GRP |
| FBLIM1 | ACACB |
| EZH2 | STXBP1 |
| ATP11A | ANKRD30A |
| PLEKHB1 | DLC1 |
| MUC16 | LINC00312 |
| FAS | NOVA1 |
| CNKSR3 | RORC |
| CSTF3 | DLG5 |
| BORA | PLA2G16 |
| COMMD2 | FOSB |
| CHEK1 | GFRA1 |
| RASD2 | SEMA3F |
| KIF11 | PCBP2 |
| SKP2 | CPE |
| KIF18B | COQ4 |
| USP10 | ELP2 |
| KNSTRN | NHSL2 |
| VSNL1 | TGFB1I1 |
| TAP1 | KIAA1462 |
| ECE2 | LOC102724275 |
| FOLR1 | CLEC11A |
| PTPN20 | DCN |
| EMG1 | ZNF703 |
| ARMC8 | PLA2G12A |
| KIFC1 | SLIT2 |
| DCLRE1B | CX3CR1 |
| DSC2 | FZD4 |
| MYO10 | FOXP1-IT1 |
| MASTL | BNC2 |
| DBF4 | ETV1 |
| RIPK2 | CYBRD1 |
| STOX2 | TFF1 |
| VGLL1 | PLEKHA6 |
| ARNTL2 | PHLDB2 |
| TMPO | SERPINA5 |
| SLC43A3 | AMIGO2 |
| ABTB2 | EVL |
| VANGL2 | ITGB5 |
| RIF1 | LNX1 |
| NAA50 | GULP1 |
| EGLN1 | CFD |
| PHF19 | RNASE4 |
| ST3GAL6 | CYP4B1 |
| SLAMF8 | YPEL2 |
| SPIN4 | UGT2B28 |
| SLC36A4 | AGR2 |
| NOP16 | ZNF224 |
| FBXO5 | FAM174B |
| OXR1 | MIR1282///HYPK///SERF2 |
| WDR4 | WLS |
| CENPBD1 | AFF3 |
| TUB | MYB |
| CENPA | NPDC1 |
| CDCA7 | PTGER3 |
| ANKRD36BP2 | ERBB4 |
| SFN | CXCL14 |
| CDC20 | RGMB |
| CENPN | EPB41L4A |
| E2F5 | LAMA2 |
| GART | PTEN |
| PELI1 | TMEM204 |
| PLK4 | NUDT4P1///NUDT4 |
| RFC4 | POLI |
| NUP50 | MMRN2 |
| S100A8 | AGR3 |
| BUB1 | IFT46 |
| POLQ | SYNPO2 |
| TCAIM | NR2F1 |
| OASL | JAM3 |
| SEH1L | ZNF559-ZNF177///ZNF177 |
| IFRD1 | PNPLA4 |
| PABPC4L | CLDN11 |
| PRR11 | TIMP3 |
| PRNP | MXRA8 |
| SKA1 | ZNF704 |
| TRIP13 | SNURF///SNRPN |
| LOC101928615///FNDC3B | PLN |
| HMGB2 | DACH1 |
| CDC7 | MRVI1 |
| LOC101928195///LOC100996643///MTHFD1L | RERG |
| NUP210 | UBE2I |
| CNOT9 | MEIS3P1 |
| DHX9 | FYCO1 |
| SKA3 | C14orf28 |
| GNL3L | GREB1 |
| GTPBP8 | GOLGA2P5 |
| SGPP2 | TRIM3 |
| CD163 | ALDH4A1 |
| MIR664B///SNORA56///DKC1 | SLC40A1 |
| ACTL8 | LINC00849///SLC25A16 |
| RFWD3 | CPLX1 |
| SMC2 | LRRC17 |
| DEDD | COPZ2 |
| MIR181A2HG | DCLK1 |
| DSC3 | GPCPD1 |
| RCCD1 | HMCN1 |
| UBE2S | LOC286191 |
| LYN | AZGP1P1///AZGP1 |
| SNRPA1 | HOXB3 |
| ID4 | TSC22D3 |
| IL12RB2 | CFH |
| STIL | PTPRB |
| UBE2C | LINC01116 |
| TFDP1 | DEGS2 |
| MND1 | ATP7A |
| HCCS | CCNG2 |
| GTPBP4 | ORMDL3 |
| GINS4 | PAN2 |
| SLC9A6 | CBR4 |
| DCUN1D1 | RBMS2 |
| IFI44L | MPV17L |
| PSAT1 | MAPT |
| FIGNL1 | RASEF |
| CCL8 | CROT |
| APH1A | EGR1 |
| CCDC82 | SLC12A2 |
| LEMD1 | STON2 |
| HTATIP2 | C1orf21 |
| TFAP2C | TOM1L2 |
| ULBP2 | ZCCHC24 |
| RRM2 | DNALI1 |
| SLC35F6///CENPA | FAAH |
| TOP2A | ASPH |
| FEN1 | TCEA2 |
| MSANTD3-TMEFF1///TMEFF1 | TM7SF2 |
| CEBPG | LTBP2 |
| HPRT1 | ABCA8 |
| HELLS | EPS8L1 |
| FANCI | STEAP4 |
| BAG2 | AOC3 |
| THNSL1 | RALGAPA2 |
| RAD54L | ZFHX4 |
| NCAPD2 | LIMA1 |
| CXCL1 | RAB27B |
| WNK3 | FBLN1 |
| PKMYT1 | TMTC3 |
| KIF18A | ANO1 |
| C21orf91 | FRMD6 |
| RTP4 | CNRIP1 |
| FAM210A | RSPH1 |
| RHBDF2 | ANK2 |
| RHBDL2 | SPON1 |
| PPAT | MIR100HG |
| LYNX1 | LMOD1 |
| FAM83D | ZBTB16 |
| NHSL1 | ABCC3 |
| CDC123 | CCL15-CCL14///CCL14 |
| MSLN | MAML3 |
| WWTR1 | MAN1C1 |
| MDN1 | LIN7A |
| PAICS | SLC25A29 |
| KLK10 | CCDC74B///CCDC74A |
| PHF20L1 | ITPR1 |
| ELF5 | SLC44A1 |
| RBM8A | CYB561D2 |
| CHAF1B | EBF1 |
| QSER1 | SH3GLB2 |
| DESI2 | ABI3BP |
| LOC100507424///ITFG2 | COL4A3BP |
| MCM6 | HTRA3 |
| TSR1 | SFRP4 |
| DPY19L4 | AGTR1 |
| NDUFA9 | AQR |
| FZD6 | PCGF2 |
| RRAGD | USP53 |
| UCHL5 | PER2 |
| AIM2 | DNAJC12 |
| SHC4 | FKBP1B |
| PPP1R14C | CXXC5 |
| FOXD1 | ADSSL1 |
| LCN2 | GLI3 |
| PRAME | CAMK2N1 |
| UHRF1 | THRSP |
| CDKN2A | C4B_2///C4B///C4A |
| SGO2 | MST1 |
| RECQL4 | FBN1 |
| MFHAS1 | INHBB |
| KIF3C | MTUS1 |
| CDKN3 | SCGB2A1 |
| FJX1 | ZG16B |
| E2F3 | DYNLRB2 |
| NUDCD1 | VPS13D |
| NDC1 | EGR3 |
| CELF4 | TSPAN5 |
| LINC01296///DUXAP10 | SLC7A8 |
| CDKAL1 | SPATA7 |
| RMI2 | CPED1 |
| MROH1 | RAB17 |
| SLC18B1 | ECI2 |
| USP1 | PRICKLE2 |
| CENPM | IVD |
| BTG3 | LYPD6 |
| RBL1 | SLC7A2 |
| APP | EIF4B |
| LOC730101 | RPS16P5 |
| FAM64A | COL3A1 |
| CENPF | WBP1L |
| PATL1 | SIGIRR |
| KRT23 | DPT |
| PFDN2 | RGS5 |
| CCDC88A | PDGFRL |
| KLHL24 | MFAP4 |
| S100A9 | TMEM150C |
| CBR1 | RARA |
| BIRC3 | LOC400043 |
| RNGTT | ECM2 |
| CDCA8 | TPRG1 |
| BYSL | MGLL |
| NUP155 | ALOX15B |
| CYP39A1 | CYP4X1 |
| TPX2 | PLK2 |
| DDX11 | CAPN13 |
| IGF2BP3 | LYRM9 |
| NDUFAF4 | ACADSB |
| RCL1 | DUSP6 |
| ANP32E | TGFB3 |
| RIOK1 | INPP4B |
| PUM3 | NAP1L3 |
| GINS1 | FAM198B |
| STK26 | TCN1 |
| NASP | SEPP1 |
| TLR1 | CPEB2 |
| KPNA2 | LOC101926921///DAB2 |
| DSCC1 | STARD10 |
| TAF5 | EXOSC7///CLEC3B |
| ANLN | PRKG1 |
| BIRC5 | UBL3 |
| TRA2B | MBD6 |
| MAGOHB///MAGOH | ACKR1 |
| A2ML1 | GUCY1A2 |
| TPI1 | PABPC1L |
| MSH6 | DNMBP |
| LAMP3 | AXIN2 |
| PLIN2 | CYP4Z1 |
| RACGAP1 | REEP5 |
| PRPS2 | FOXA1 |
| MRPS12 | ZNF844 |
| RYR1 | SLC1A4 |
| PHLDA1 | PTPN21 |
| SUV39H2 | SAP18 |
| PALM2 | LIMCH1 |
| HINT3 | TSHZ3 |
| C12orf4 | CCDC80 |
| CDC6 | SPDEF |
| LOC642846///DDX12P///DDX11 | AZGP1 |
| CHI3L1 | CLU |
| HMGB3P1 | CXCL12 |
| WDR12 | SRSF1 |
| APOBEC3B | 2-Mar |
| AURKA | LDB2 |
| IL27RA | C16orf89 |
| IGLV1-44 | IGF1R |
| RBM38 | COL14A1 |
| ALYREF | PGAP3 |
| AURKB | EFEMP2 |
| TIPIN | SCGB2A2 |
| RASSF4 | CSAD |
| DEF8 | RECK |
| CHAF1A | ABAT |
| MUC5B | NAV1 |
| WFDC21P | IRX2 |
| TMEM170A | JAM2 |
| CCDC77 | SPARCL1 |
| FANCA | IL6ST |
| PDSS1 | FAM172A |
| LINC00960 | SPATA20 |
| AGO2 | STEAP2 |
| C5orf46 | RABEP1 |
| HAUS7///TREX2 | C19orf33 |
| RAD54B | BICC1 |
| MSANTD3 | NR2F1-AS1 |
| SPP1 | SMIM14 |
| MIR3658///UCK2 | SERHL2 |
| BARD1 | ADCY6 |
| SPDL1 | LOC102725051///LOC102723891///ANKRD20A4///ANKRD20A2///ANKRD20A3///ANKRD20A5P///ANKRD20A11P///ANKRD20A9P///ANKRD20A1 |
| RANBP1 | GSTM3 |
| CNOT7 | DST |
| C1GALT1 | DR1 |
| IL4I1 | XBP1 |
| SMCO4 | SLC46A1 |
| IMPA2 | COX7A1 |
| PSIP1 | ACER2 |
| SMCHD1 | CMBL |
| TEX10 | KIF13B |
| EPHB3 | RAB30 |
| TSPYL5 | ZNF626 |
| ITGB8 | CEP126 |
| TPD52L1 | SOCS2 |
| PAK1IP1 | RBMS3 |
| SLC7A11 | MINOS1-NBL1///NBL1 |
| NAA15 | LLPH |
| FRMD4A | CRACR2A |
| GTSE1 | GAS7 |
| MRPL9 | MEG3 |
| YES1 | MEST |
| NUF2 | ADH1B |
| TRMT11 | ANGPTL2 |
| STAT1 | RAB11A |
| PTTG1 | SCUBE2 |
| NRTN | PIP |
| MMP12 | ENPP1 |
| CDCA5 | SCNN1A |
| UBE2T | FABP4 |
| SLC2A1 | MIR631///NEIL1 |
| CREM | IDUA |
| CHMP4C | PDZK1 |
| BCL2A1 | PEX11A |
| TEAD4 | CIRBP |
| ECT2 | SYT17 |
| PTDSS1 | FRZB |
| HACD1 | FOXP1 |
| CDH3 | ZFHX3 |
| IQGAP3 | HSD17B8 |
| MYBL1 | SHISA2 |
| PDZK1IP1 | PPP1R3C |
| VASH2 | TAPT1 |
| COL9A3 | ARL15 |
| DIAPH3 | HIVEP3 |
| GGH | MIR4800///MXD4 |
| ME2 | ABLIM3 |
| KPNA4 | SEMA5A |
| RAP2B///RAP2A | STC2 |
| KIAA1549///BRAF | NUMA1 |
| MIR6836///SNX8 | KITLG |
| EFNA5 | SUPT6H |
| TMEM68 | ELL2 |
| MTFR2 | VIPR1 |
| CENPE | SPARC |
| ACTR3B | THSD4 |
| CMSS1 | IGFBP4 |
| PLCH1 | HOXC6 |
| LINC00673///LINC00511 | TMEM119 |
| TACC3 | NME3 |
| FAM57A | SNED1 |
| YRDC | LPAR1 |
| MEX3A | ACOX2 |
| CMC2 | MLPH |
| FSCN1 | RETSAT |
| MCM7 | FAM110B |
| SRD5A1 | TBC1D9 |
| CLEC7A | PHYHD1 |
| HSPA14 | GAB1 |
| PSMG1 | RBM43 |
| GBP1 | AR |
| CEP55 | PLXDC1 |
| PLSCR1 | EPB41L5 |
| PLEKHG4B | KLHL3 |
| CTPS1 | TFF3 |
| LAD1 | ADCY9 |
| SYNCRIP | PTPN13 |
| MTAP | SUOX |
| GZMB | OLFML1 |
| TMEM38B | DAAM1 |
| PDE7A | SELENBP1 |
| NT5DC2 | CRACR2B |
| CYBB | GSTT1 |
| XPNPEP3 | TNS2 |
| PMAIP1 | DNAH5 |
| EIF4EBP1 | MTERF2 |
| PLA2G7 | SEMA3C |
| SUSD4 | DPYSL3 |
| COA7 | DYX1C1-CCPG1///CCPG1 |
| TTC22 | ATP2B1 |
| JMJD4 | LOC100507577///LONP2///SIAH1 |
| DCPS | NDN |
| DLGAP5 | LOC100506718///FLRT2 |
| RAD51AP1 | OSBPL5 |
| MIR7112///BOP1 | DHRS2 |
| TMEM108 | SLC39A6 |
| ENO1 |  |
| MKI67 |  |
| SLC39A8 |  |
| GLS |  |
| CCL5 |  |
| TM4SF1 |  |
| MX1 |  |
| RELB |  |
| IL32 |  |
| ELAVL1 |  |
| SLC35F2 |  |
| C9orf40 |  |
| NMU |  |
| ASXL1 |  |
| IFIH1 |  |
| AZI2 |  |
| NUP205 |  |
| PLEKHG1 |  |
| GABBR2 |  |
| AGBL5 |  |
| CEACAM1 |  |
| BEND3 |  |
| LPIN1 |  |
| NXT2 |  |
| CDC42EP1 |  |
| ME1 |  |
| COL27A1 |  |
| ZNF883 |  |
| FBXL13 |  |
| SLC16A1 |  |
| C4orf46 |  |
| SMIM13 |  |
| TP53BP2 |  |
| GINS2 |  |
| PDLIM5 |  |
| MOB3B |  |
| RPL39L |  |
| DONSON |  |
| E2F8 |  |
| IQCJ-SCHIP1///SCHIP1 |  |
| SOS1 |  |
| MPZL2 |  |
| DNAH14 |  |
| FRMD3 |  |
| COL22A1 |  |
| MATR3 |  |
| UTP4 |  |
| HMGB3 |  |
| AFAP1-AS1 |  |
| RFC3 |  |
| ILF2 |  |
| GPX7 |  |
| ARHGEF7 |  |
| ADAMDEC1 |  |
| CALML4 |  |
| UCHL1 |  |
| APOL6 |  |
| PLK1 |  |
| KLHL23 |  |
| PM20D2 |  |
| TFCP2L1 |  |
| STEAP3 |  |
| SDR42E1 |  |
| NOP2 |  |
| FAM216A |  |
| CTSV |  |
| KLHDC7B |  |
| HAUS6 |  |
| PRKD3 |  |
| CDC25C |  |
| LAG3 |  |
| DSG2 |  |
| CXCL11 |  |
| ZNF639 |  |
| MYBL2 |  |
| HSPA4L |  |
| SMAGP |  |
| SMC4 |  |
| HLA-DQB1 |  |
| SCRG1 |  |
| NRAS |  |
| CENPO |  |
| CXCL8 |  |
| SIX3 |  |
| SIMC1 |  |
| PFN2 |  |
| CKS1B |  |
| MCM3 |  |
| USP13 |  |
| FAF1 |  |
| CDK6 |  |
| IGHM |  |
| H2BFXP |  |
| ZIC1 |  |
| RHNO1 |  |
| ST8SIA1 |  |
| GAS2L3 |  |
| BLM |  |
| PSMB2 |  |
| WARS |  |
| SNRPD1 |  |
| NCAPG2 |  |
| FGFR2 |  |
| ATG5 |  |
| EPRS |  |
| HR |  |
| CCL20 |  |
| PRKX |  |
| KIF2C |  |
| PRPS1 |  |
| CCSAP |  |
| DIEXF |  |
| ZNF644 |  |
| IQCG |  |
| PDCD2L |  |
| COQ3 |  |
| MTBP |  |
| TFRC |  |
| AK2 |  |
| NANP |  |
| ZFP82 |  |
| PBK |  |
| MB21D1 |  |
| FAM26F |  |
| CHMP4B |  |
| MED30 |  |
| MKRN2 |  |
| PSME4 |  |
| TLDC1 |  |
| DUXAP10 |  |
| MTPAP |  |
| RPF2 |  |
| FAM60A |  |
| DNAJC6 |  |
| SLCO4A1 |  |
| NCAPH |  |
| PTPRK |  |
| TK1 |  |
| HLA-F |  |
| MOXD1 |  |
| NIP7 |  |
| TMEM158 |  |
| ANKRD16 |  |
| SHCBP1 |  |
| COTL1 |  |
| ARL9 |  |
| ASPM |  |
| TLE1 |  |
| PPARA |  |
| PRIM1 |  |
| CEP78 |  |
| CDCA3 |  |
| KNL1 |  |
| ATAD2 |  |
| BRIP1 |  |
| RASGRP1 |  |
| SLC7A5 |  |
| NFIB |  |
| CT83 |  |
| TMSB15B///TMSB15A |  |
| NLRP2 |  |
| FOXP4-AS1 |  |
| POLE2 |  |
| ENY2 |  |
| TNFSF13B |  |
| SAP30 |  |
| CKAP2 |  |
| AUNIP |  |
| KHDRBS3 |  |
| ICAM1 |  |
| EGFL6 |  |
| ZNF367 |  |
| HDAC2 |  |
| ETS1 |  |
| FGD1 |  |
| UBAP2L |  |
| SPC25 |  |
| SLC25A33 |  |
| PAPSS1 |  |
| KIF15 |  |
| MSH2 |  |
| DSG3 |  |
| BUB1B |  |
| HJURP |  |
| RNASEH2A |  |
| PRPF38A |  |
| DTL |  |
| ATP6V1C2 |  |
| CENPV |  |
| MIR6756///MCAM |  |
| PDK1 |  |
| RAD51 |  |
| FERMT1 |  |
| TMEM30A |  |
| HMMR |  |
| LY6E |  |
| CDK19 |  |
| CCDC150 |  |
| ART3 |  |
| KIAA1804 |  |
| C16orf95 |  |
| GMNN |  |
| CASC7///AGO2 |  |
| KIF20A |  |
| YBX3 |  |
| RAD21 |  |
| ORC6 |  |
| SOX11 |  |
| CEP152 |  |
| NUDT5 |  |
| BCL11A |  |
| GMPS |  |
| GPT2 |  |
| ZHX1-C8orf76 |  |
| ZNF286B///ZNF286A |  |
| KCNK5 |  |
| TEX30 |  |
| LARP4B |  |
| ETV6 |  |
| CHAC2 |  |
| CEBPB |  |
| GATAD2B |  |
| FMNL2 |  |
| HMGA1 |  |
| CXCL16 |  |
| STRN |  |
| TTK |  |
| NCAPG |  |
| TIFA |  |
| CD274 |  |
| CP |  |
| AMD1 |  |
| NUSAP1 |  |
| CIART |  |
| DCLRE1C |  |
| PSMB9 |  |
| XPO5 |  |
| CTSC |  |
