# Supplementary material for: Construction of an mRNA-miRNA-lncRNA network prognostic for triple-negative breast cancer
Source: Aging (Albany NY). 2021 Jan 3;13(1):1153–75. doi: 10.18632/aging.202254 (PMC7835059; doi:10.18632/aging.202254)
Supplement: Supplementary Tables 2 and 3 [file aging-13-202254-s003.pdf]

Supplementary Table 2. The expression of 12 hub genes in TNBC from TCGA.

Gene symbol	logFC	*P*	Adjusted *P*
CDK1	3.72	1.51e-21	1.22e-18
CCNB1	2.83	4.95e-19	2.30e-16
CCNA2	3.65	6.00e-20	3.30e-17
CDC20	4.75	1.31e-26	6.04e-23
TOP2A	4.18	1.90e-19	9.41e-17
CCNB2	3.92	2.09e-23	2.81e-20
MAD2L1	2.51	3.93e-11	3.44e-09
BUB1	4.39	8.99e-23	1.01e-19
KIF11	3.09	6.10e-17	1.84e-14
RRM2	4.41	1.66e-26	6.71e-23
ESR1	–4.79	5.11e-14	8.00e-12
IGF1R	–1.34	0.00754	0.046499

Supplementary Table 3. The mRNA-miRNA pairs predicted by miRTarBase.

mRNA	miRNA
CCNA2	hsa-let-7b-5p
CCNA2	hsa-miR-10b-3p
CCNA2	hsa-miR-130b-3p
CCNA2	hsa-miR-27b-3p
CCNA2	hsa-miR-22-3p
MAD2L1	hsa-miR-192-5p
MAD2L1	hsa-miR-28-5p
CDK1	hsa-miR-31-5p
CDK1	hsa-miR-24-3p
RRM2	hsa-let-7a-5p
CCNB1	hsa-miR-410-3p
ESR1	hsa-miR-302c-3p
ESR1	hsa-miR-206
ESR1	hsa-miR-193b-3p
ESR1	hsa-miR-18b-5p
ESR1	hsa-miR-22-3p
ESR1	hsa-miR-19a-3p
ESR1	hsa-miR-19b-3p
ESR1	hsa-miR-20b-5p
ESR1	hsa-miR-221-3p
ESR1	hsa-miR-222-3p
ESR1	hsa-miR-130a-3p
ESR1	hsa-miR-26a-5p
ESR1	hsa-miR-145-5p
ESR1	hsa-miR-26b-3p
ESR1	hsa-miR-192-5p
IGF1	hsa-miR-1-3p
IGF1	hsa-miR-27a-3p
IGF1	hsa-miR-483-3p
IGF1	hsa-let-7e-5p
IGF1	hsa-miR-190b
IGF1	hsa-let-7i-5p
IGF1	hsa-miR-199a-3p
IGF1	hsa-miR-190a-5p
IGF1	hsa-miR-29a-3p
IGF1	hsa-miR-133a-3p
IGF1	hsa-miR-18b-5p
IGF1	hsa-miR-28-5p
IGF1	hsa-miR-603

IGF1	hsa-miR-15b-3p
IGF1	hsa-miR-130b-3p
IGF1	hsa-miR-98-5p
IGF1	hsa-miR-26b-5p
IGF1	hsa-miR-129-5p
IGF1	hsa-miR-128-3p
IGF1	hsa-miR-26a-5p
