# Supplementary material for: Construction of an mRNA-miRNA-lncRNA network prognostic for triple-negative breast cancer
Source: Aging (Albany NY). 2021 Jan 3;13(1):1153–75. doi: 10.18632/aging.202254 (PMC7835059; doi:10.18632/aging.202254)
Supplement: Supplementary Table 4 [file aging-13-202254-s004.docx]

**Supplementary Table 4. The lncRNA-miRNA pairs predicted by miRNet database.**

| **miRNA** | **lncRNA** |
| --- | --- |
| hsa-let-7a-5p | XIST |
| hsa-let-7b-5p | XIST |
| hsa-let-7a-5p | UBXN8 |
| hsa-let-7b-5p | UBXN8 |
| hsa-let-7a-5p | ZNF518A |
| hsa-let-7b-5p | ZNF518A |
| hsa-let-7a-5p | CASP8AP2 |
| hsa-let-7b-5p | CASP8AP2 |
| hsa-let-7a-5p | KCNQ1OT1 |
| hsa-let-7b-5p | KCNQ1OT1 |
| hsa-let-7a-5p | TTTY15 |
| hsa-let-7b-5p | TTTY15 |
| hsa-let-7a-5p | LINC00263 |
| hsa-let-7b-5p | LINC00263 |
| hsa-let-7a-5p | MAL2 |
| hsa-let-7b-5p | MAL2 |
| hsa-let-7a-5p | NEAT1 |
| hsa-let-7b-5p | NEAT1 |
| hsa-let-7a-5p | SCARNA10 |
| hsa-let-7b-5p | SCARNA10 |
| hsa-let-7a-5p | CDKN2B-AS1 |
| hsa-let-7b-5p | CDKN2B-AS1 |
| hsa-let-7a-5p | MIR4720 |
| hsa-let-7b-5p | MIR4720 |
| hsa-let-7a-5p | AC074117.10 |
| hsa-let-7a-5p | AC105760.2 |
| hsa-let-7a-5p | CTA-204B4.6 |
| hsa-let-7a-5p | CTD-2270L9.5 |
| hsa-let-7a-5p | CTD-3138B18.5 |
| hsa-let-7a-5p | HOXA11-AS |
| hsa-let-7a-5p | hsa-mir-6080 |
| hsa-let-7a-5p | LINC00665 |
| hsa-let-7a-5p | NUTM2A-AS1 |
| hsa-let-7a-5p | PTCHD3P1 |
| hsa-let-7a-5p | RP11-1103G16.1 |
| hsa-let-7a-5p | RP11-197N18.2 |
| hsa-let-7a-5p | RP11-214O1.2 |
| hsa-let-7a-5p | RP11-264B17.3 |
| hsa-let-7a-5p | RP11-277P12.20 |
| hsa-let-7a-5p | RP11-27I1.2 |
| hsa-let-7a-5p | RP11-282O18.6 |
| hsa-let-7a-5p | RP11-452L6.5 |
| hsa-let-7a-5p | RP11-457M11.2 |
| hsa-let-7a-5p | RP11-473I1.10 |
| hsa-let-7a-5p | RP11-492E3.1 |
| hsa-let-7a-5p | RP11-498P14.3 |
| hsa-let-7a-5p | RP11-727A23.5 |
| hsa-let-7a-5p | RP11-819C21.1 |
| hsa-let-7a-5p | RP11-834C11.4 |
| hsa-let-7a-5p | RP11-923I11.1 |
| hsa-let-7a-5p | RP11-996F15.2 |
| hsa-let-7a-5p | RP13-507I23.1 |
| hsa-let-7a-5p | SNHG16 |
| hsa-let-7a-5p | XXbac-BPG300A18.13 |
| hsa-let-7b-5p | AC074117.10 |
| hsa-let-7b-5p | AC105760.2 |
| hsa-let-7b-5p | CTA-204B4.6 |
| hsa-let-7b-5p | CTD-2270L9.5 |
| hsa-let-7b-5p | CTD-3138B18.5 |
| hsa-let-7b-5p | HOXA11-AS |
| hsa-let-7b-5p | hsa-mir-6080 |
| hsa-let-7b-5p | LINC00665 |
| hsa-let-7b-5p | NUTM2A-AS1 |
| hsa-let-7b-5p | PTCHD3P1 |
| hsa-let-7b-5p | RP11-1103G16.1 |
| hsa-let-7b-5p | RP11-197N18.2 |
| hsa-let-7b-5p | RP11-214O1.2 |
| hsa-let-7b-5p | RP11-264B17.3 |
| hsa-let-7b-5p | RP11-277P12.20 |
| hsa-let-7b-5p | RP11-27I1.2 |
| hsa-let-7b-5p | RP11-282O18.6 |
| hsa-let-7b-5p | RP11-452L6.5 |
| hsa-let-7b-5p | RP11-457M11.2 |
| hsa-let-7b-5p | RP11-473I1.10 |
| hsa-let-7b-5p | RP11-492E3.1 |
| hsa-let-7b-5p | RP11-498P14.3 |
| hsa-let-7b-5p | RP11-727A23.5 |
| hsa-let-7b-5p | RP11-819C21.1 |
| hsa-let-7b-5p | RP11-834C11.4 |
| hsa-let-7b-5p | RP11-923I11.1 |
| hsa-let-7b-5p | RP11-996F15.2 |
| hsa-let-7b-5p | RP13-507I23.1 |
| hsa-let-7b-5p | SNHG16 |
| hsa-let-7b-5p | XXbac-BPG300A18.13 |
| hsa-mir-410-3p | XIST |
| hsa-mir-410-3p | SCAMP1 |
| hsa-mir-410-3p | TUG1 |
| hsa-mir-410-3p | MAL2 |
| hsa-mir-410-3p | C17orf76-AS1 |
| hsa-mir-410-3p | OIP5-AS1 |
| hsa-mir-410-3p | AC005562.1 |
| hsa-mir-410-3p | AC016629.8 |
| hsa-mir-410-3p | CCAT1 |
| hsa-mir-410-3p | CTA-217C2.1 |
| hsa-mir-410-3p | LINC00657 |
| hsa-mir-410-3p | RP1-228H13.5 |
| hsa-mir-410-3p | RP11-119F7.5 |
| hsa-mir-410-3p | RP11-175O19.4 |
| hsa-mir-410-3p | RP11-18I14.10 |
| hsa-mir-410-3p | RP11-204M4.2 |
| hsa-mir-410-3p | RP11-398F12.1 |
| hsa-mir-410-3p | RP11-473I1.9 |
| hsa-mir-410-3p | RP11-566E18.3 |
| hsa-mir-410-3p | RP11-686D22.8 |
| hsa-mir-410-3p | RP11-738E22.2 |
| hsa-mir-410-3p | RP11-758M4.4 |
| hsa-mir-410-3p | RP6-24A23.7 |
| hsa-let-7e-5p | XIST |
| hsa-mir-19a-3p | XIST |
| hsa-mir-98-5p | XIST |
| hsa-mir-130b-3p | XIST |
| hsa-mir-18b-5p | XIST |
| hsa-let-7e-5p | UBXN8 |
| hsa-mir-98-5p | UBXN8 |
| hsa-mir-19a-3p | JRK |
| hsa-mir-19a-3p | MAP3K14 |
| hsa-mir-130b-3p | MAP3K14 |
| hsa-mir-18b-5p | RECQL4 |
| hsa-mir-19a-3p | SEC22B |
| hsa-mir-130b-3p | SEC22B |
| hsa-let-7e-5p | ZNF518A |
| hsa-mir-19a-3p | ZNF518A |
| hsa-mir-98-5p | ZNF518A |
| hsa-let-7e-5p | CASP8AP2 |
| hsa-mir-98-5p | CASP8AP2 |
| hsa-let-7e-5p | KCNQ1OT1 |
| hsa-mir-19a-3p | KCNQ1OT1 |
| hsa-mir-98-5p | KCNQ1OT1 |
| hsa-mir-130b-3p | KCNQ1OT1 |
| hsa-mir-18b-5p | KCNQ1OT1 |
| hsa-mir-18b-5p | SNHG1 |
| hsa-mir-18b-5p | POLDIP2 |
| hsa-mir-19a-3p | LINC00339 |
| hsa-mir-130b-3p | LINC00339 |
| hsa-mir-222-3p | TUG1 |
| hsa-mir-222-3p | GAS5 |
| hsa-mir-18b-5p | GAS5 |
| hsa-let-7e-5p | TTTY15 |
| hsa-mir-98-5p | TTTY15 |
| hsa-mir-19a-3p | ZNRD1-AS1 |
| hsa-mir-130b-3p | ZNRD1-AS1 |
| hsa-mir-18b-5p | LINC00467 |
| hsa-mir-19a-3p | CROCCP2 |
| hsa-let-7e-5p | LINC00263 |
| hsa-mir-98-5p | LINC00263 |
| hsa-mir-19a-3p | LINC00313 |
| hsa-mir-19a-3p | MCM3AP-AS1 |
| hsa-let-7e-5p | MAL2 |
| hsa-mir-19a-3p | MAL2 |
| hsa-mir-98-5p | MAL2 |
| hsa-mir-19a-3p | LINC00342 |
| hsa-mir-130b-3p | LINC00116 |
| hsa-mir-18b-5p | CASC2 |
| hsa-mir-19a-3p | ZNF718 |
| hsa-mir-19a-3p | LINC00094 |
| hsa-mir-19a-3p | H19 |
| hsa-mir-130b-3p | H19 |
| hsa-mir-18b-5p | H19 |
| hsa-let-7e-5p | NEAT1 |
| hsa-mir-98-5p | NEAT1 |
| hsa-mir-18b-5p | SNHG15 |
| hsa-mir-19a-3p | HOXD-AS1 |
| hsa-mir-130b-3p | HOXD-AS1 |
| hsa-mir-130b-3p | MIR17HG |
| hsa-mir-19a-3p | LINC00338 |
| hsa-let-7e-5p | SCARNA10 |
| hsa-mir-98-5p | SCARNA10 |
| hsa-mir-18b-5p | OIP5-AS1 |
| hsa-let-7e-5p | CDKN2B-AS1 |
| hsa-mir-98-5p | CDKN2B-AS1 |
| hsa-mir-19a-3p | HOTAIR |
| hsa-mir-222-3p | HOTAIR |
| hsa-mir-130b-3p | HOTAIR |
| hsa-mir-19a-3p | FGD5-AS1 |
| hsa-mir-130b-3p | FGD5-AS1 |
| hsa-mir-130b-3p | BOLA3-AS1 |
| hsa-let-7e-5p | MIR4720 |
| hsa-mir-98-5p | MIR4720 |
| hsa-let-7e-5p | AC074117.10 |
| hsa-let-7e-5p | AC105760.2 |
| hsa-let-7e-5p | CTA-204B4.6 |
| hsa-let-7e-5p | CTD-2270L9.5 |
| hsa-let-7e-5p | CTD-3138B18.5 |
| hsa-let-7e-5p | HOXA11-AS |
| hsa-let-7e-5p | hsa-mir-6080 |
| hsa-let-7e-5p | LINC00665 |
| hsa-let-7e-5p | NUTM2A-AS1 |
| hsa-let-7e-5p | PTCHD3P1 |
| hsa-let-7e-5p | RP11-1103G16.1 |
| hsa-let-7e-5p | RP11-197N18.2 |
| hsa-let-7e-5p | RP11-214O1.2 |
| hsa-let-7e-5p | RP11-264B17.3 |
| hsa-let-7e-5p | RP11-277P12.20 |
| hsa-let-7e-5p | RP11-27I1.2 |
| hsa-let-7e-5p | RP11-282O18.6 |
| hsa-let-7e-5p | RP11-452L6.5 |
| hsa-let-7e-5p | RP11-457M11.2 |
| hsa-let-7e-5p | RP11-473I1.10 |
| hsa-let-7e-5p | RP11-492E3.1 |
| hsa-let-7e-5p | RP11-498P14.3 |
| hsa-let-7e-5p | RP11-727A23.5 |
| hsa-let-7e-5p | RP11-819C21.1 |
| hsa-let-7e-5p | RP11-834C11.4 |
| hsa-let-7e-5p | RP11-923I11.1 |
| hsa-let-7e-5p | RP11-996F15.2 |
| hsa-let-7e-5p | RP13-507I23.1 |
| hsa-let-7e-5p | SNHG16 |
| hsa-let-7e-5p | XXbac-BPG300A18.13 |
| hsa-mir-19a-3p | AC005307.3 |
| hsa-mir-19a-3p | AC006445.8 |
| hsa-mir-19a-3p | AC007038.7 |
| hsa-mir-19a-3p | AC079767.4 |
| hsa-mir-19a-3p | AC135048.13 |
| hsa-mir-19a-3p | AL589743.1 |
| hsa-mir-19a-3p | AP001065.15 |
| hsa-mir-19a-3p | CTA-204B4.6 |
| hsa-mir-19a-3p | CTB-50L17.7 |
| hsa-mir-19a-3p | CTB-89H12.4 |
| hsa-mir-19a-3p | CTB-92J24.2 |
| hsa-mir-19a-3p | CTC-479C5.16 |
| hsa-mir-19a-3p | CTD-2369P2.2 |
| hsa-mir-19a-3p | HOXA-AS4 |
| hsa-mir-19a-3p | LINC00667 |
| hsa-mir-19a-3p | LINC00839 |
| hsa-mir-19a-3p | PRKCQ-AS1 |
| hsa-mir-19a-3p | RP1-178F10.3 |
| hsa-mir-19a-3p | RP11-115C21.2 |
| hsa-mir-19a-3p | RP11-139H15.1 |
| hsa-mir-19a-3p | RP11-170L3.8 |
| hsa-mir-19a-3p | RP11-197P3.5 |
| hsa-mir-19a-3p | RP11-206L10.11 |
| hsa-mir-19a-3p | RP11-218M22.1 |
| hsa-mir-19a-3p | RP11-290D2.4 |
| hsa-mir-19a-3p | RP11-355O1.11 |
| hsa-mir-19a-3p | RP11-363E7.4 |
| hsa-mir-19a-3p | RP11-363G2.4 |
| hsa-mir-19a-3p | RP11-399K21.11 |
| hsa-mir-19a-3p | RP11-412D9.4 |
| hsa-mir-19a-3p | RP11-418J17.1 |
| hsa-mir-19a-3p | RP11-429J17.6 |
| hsa-mir-19a-3p | RP11-46C20.1 |
| hsa-mir-19a-3p | RP11-588K22.2 |
| hsa-mir-19a-3p | RP11-690D19.3 |
| hsa-mir-19a-3p | RP11-73M18.8 |
| hsa-mir-19a-3p | RP11-758M4.4 |
| hsa-mir-19a-3p | RP11-819C21.1 |
| hsa-mir-19a-3p | RP11-84C13.1 |
| hsa-mir-19a-3p | RP3-523K23.2 |
| hsa-mir-19a-3p | SLC26A4-AS1 |
| hsa-mir-19a-3p | TM4SF19-AS1 |
| hsa-mir-19a-3p | XXbac-B461K10.4 |
| hsa-mir-98-5p | AC074117.10 |
| hsa-mir-98-5p | AC105760.2 |
| hsa-mir-98-5p | CTA-204B4.6 |
| hsa-mir-98-5p | CTD-2270L9.5 |
| hsa-mir-98-5p | CTD-3138B18.5 |
| hsa-mir-98-5p | HOXA11-AS |
| hsa-mir-98-5p | hsa-mir-6080 |
| hsa-mir-98-5p | LINC00665 |
| hsa-mir-98-5p | NUTM2A-AS1 |
| hsa-mir-98-5p | PTCHD3P1 |
| hsa-mir-98-5p | RP11-1103G16.1 |
| hsa-mir-98-5p | RP11-197N18.2 |
| hsa-mir-98-5p | RP11-214O1.2 |
| hsa-mir-98-5p | RP11-264B17.3 |
| hsa-mir-98-5p | RP11-277P12.20 |
| hsa-mir-98-5p | RP11-27I1.2 |
| hsa-mir-98-5p | RP11-282O18.6 |
| hsa-mir-98-5p | RP11-452L6.5 |
| hsa-mir-98-5p | RP11-457M11.2 |
| hsa-mir-98-5p | RP11-473I1.10 |
| hsa-mir-98-5p | RP11-492E3.1 |
| hsa-mir-98-5p | RP11-498P14.3 |
| hsa-mir-98-5p | RP11-727A23.5 |
| hsa-mir-98-5p | RP11-819C21.1 |
| hsa-mir-98-5p | RP11-834C11.4 |
| hsa-mir-98-5p | RP11-923I11.1 |
| hsa-mir-98-5p | RP11-996F15.2 |
| hsa-mir-98-5p | RP13-507I23.1 |
| hsa-mir-98-5p | SNHG16 |
| hsa-mir-98-5p | XXbac-BPG300A18.13 |
| hsa-mir-222-3p | AC000120.7 |
| hsa-mir-222-3p | CTA-204B4.6 |
| hsa-mir-222-3p | CTD-3099C6.9 |
| hsa-mir-222-3p | DHRS4-AS1 |
| hsa-mir-222-3p | GS1-259H13.2 |
| hsa-mir-222-3p | hsa-mir-6080 |
| hsa-mir-222-3p | RP11-170L3.8 |
| hsa-mir-222-3p | RP11-325K4.2 |
| hsa-mir-222-3p | RP11-325K4.3 |
| hsa-mir-222-3p | RP11-372K14.2 |
| hsa-mir-222-3p | RP11-473I1.9 |
| hsa-mir-222-3p | RP11-477D19.2 |
| hsa-mir-222-3p | RP11-498E2.8 |
| hsa-mir-222-3p | RP11-529K1.2 |
| hsa-mir-222-3p | RP11-53I6.2 |
| hsa-mir-222-3p | RP11-552M11.4 |
| hsa-mir-222-3p | RP11-57H14.4 |
| hsa-mir-222-3p | RP11-819C21.1 |
| hsa-mir-222-3p | RP11-96D1.10 |
| hsa-mir-130b-3p | AC007038.7 |
| hsa-mir-130b-3p | AC007040.7 |
| hsa-mir-130b-3p | AC084018.1 |
| hsa-mir-130b-3p | AC084219.4 |
| hsa-mir-130b-3p | AC135048.13 |
| hsa-mir-130b-3p | AP001065.15 |
| hsa-mir-130b-3p | CCAT1 |
| hsa-mir-130b-3p | CTA-204B4.6 |
| hsa-mir-130b-3p | CTB-92J24.2 |
| hsa-mir-130b-3p | CTC-281B15.1 |
| hsa-mir-130b-3p | CTD-2369P2.2 |
| hsa-mir-130b-3p | CTD-3092A11.2 |
| hsa-mir-130b-3p | HOXA11-AS |
| hsa-mir-130b-3p | LINC00667 |
| hsa-mir-130b-3p | LINC00839 |
| hsa-mir-130b-3p | NUTM2A-AS1 |
| hsa-mir-130b-3p | PRKCQ-AS1 |
| hsa-mir-130b-3p | RP1-178F10.3 |
| hsa-mir-130b-3p | RP11-197P3.5 |
| hsa-mir-130b-3p | RP11-290D2.4 |
| hsa-mir-130b-3p | RP11-344B2.2 |
| hsa-mir-130b-3p | RP11-355O1.11 |
| hsa-mir-130b-3p | RP11-361F15.2 |
| hsa-mir-130b-3p | RP11-363E7.4 |
| hsa-mir-130b-3p | RP11-429J17.6 |
| hsa-mir-130b-3p | RP11-588K22.2 |
| hsa-mir-130b-3p | RP11-656D10.3 |
| hsa-mir-130b-3p | RP11-73M18.8 |
| hsa-mir-130b-3p | RP11-84C13.1 |
| hsa-mir-130b-3p | RP13-638C3.2 |
| hsa-mir-130b-3p | RP4-639F20.3 |
| hsa-mir-130b-3p | SLC26A4-AS1 |
| hsa-mir-130b-3p | TMEM161B-AS1 |
| hsa-mir-18b-5p | AC156455.1 |
| hsa-mir-18b-5p | AF011889.5 |
| hsa-mir-18b-5p | BCDIN3D-AS1 |
| hsa-mir-18b-5p | FENDRR |
| hsa-mir-18b-5p | GS1-358P8.4 |
| hsa-mir-18b-5p | hsa-mir-212 |
| hsa-mir-18b-5p | KB-318B8.7 |
| hsa-mir-18b-5p | LINC00617 |
| hsa-mir-18b-5p | LINC00622 |
| hsa-mir-18b-5p | LOXL1-AS1 |
| hsa-mir-18b-5p | RP1-37E16.12 |
| hsa-mir-18b-5p | RP11-170L3.8 |
| hsa-mir-18b-5p | RP11-175O19.4 |
| hsa-mir-18b-5p | RP11-228B15.4 |
| hsa-mir-18b-5p | RP11-311C24.1 |
| hsa-mir-18b-5p | RP11-372K14.2 |
| hsa-mir-18b-5p | RP11-379K17.11 |
| hsa-mir-18b-5p | RP11-498C9.15 |
| hsa-mir-18b-5p | RP11-553L6.5 |
| hsa-mir-18b-5p | RP11-690D19.3 |
| hsa-mir-18b-5p | RP11-834C11.4 |
| hsa-mir-18b-5p | RP11-977G19.5 |
| hsa-mir-18b-5p | RP5-894A10.6 |
| hsa-mir-18b-5p | RP6-24A23.7 |
| hsa-mir-18b-5p | SDCBP2-AS1 |
| hsa-mir-18b-5p | SPTY2D1-AS1 |
| hsa-mir-18b-5p | Z97634.5 |
